# Supplementary material for: Evaluation of NPP using three models compared with MODIS-NPP data over China
Source: PLoS One. 2021 Nov 18;16(11):e0252149. doi: 10.1371/journal.pone.0252149 (PMC8601518; doi:10.1371/journal.pone.0252149)
Supplement: Attachment — Submitted filename: PONE-D-20-31479_reviewer_Reviewed.pdf [file pone.0252149.s001.pdf]

PLOS ONE

Spatial Pattern Change and Analysis of NPP in Terrestrial Vegetation Ecosystem based on three models in China

--Manuscript Draft--

**Manuscript Number:**	PONE-D-20-31479
**Article Type:**	Research Article
**Full Title:**	Spatial Pattern Change and Analysis of NPP in Terrestrial Vegetation Ecosystem based on three models in China
**Short Title:**	Evaluation of NPP using three models compared over China
**Corresponding Author:**	Jinke Sun, ph.D<br>Henan Polytechnic University<br>JiaoZuo, HeNan CHINA
**Keywords:**	NPP estimation, climate productivity model, Synthetic model, CASA-model, MOD17A3, statistics evaluation
**Abstract:**	ABSTRACT Estimating net primary productivity (NPP) is crucial in global climate change research and carbon cycling. However, there are many uncertainties different NPP modeling results and the process ofNPP is challenging to model on the absence of data. In this study, we only used meteorological data as input, the estimated NPP using climate productivity model, synthetic model and CASA model were compared with MOD17A3-NPP as the reference data over China during the past 16 years (2000-2015). Then the statistics evaluation metrics (Relative Bias (RB), Pearson linear Correlation Coefficient (CC), Root Mean Square Error (RMSE), and Nash-Sutcliffe efficiency coefficient (NSE)) for three models with the reference data were calculated. The correlation analysis implied that the CASA-model performed better than the other two models in terms of RB, RMSE, NSE and CC whether on the national or the regional scale. It has a higher CC with 0.5081 and a smaller RMSE with 111.96 g C·m -2 ·yr -1 in the whole country. The synthetic model and CASA-model has the same advantages at some regions, and the artificial model has lower RMSE in Southern China (86.35 g C·m -2 ·yr -1), Xinjiang (85.53 g C·m -2 ·yr -1) and Qinghai-Tibet Plateau (93.22 g C·m -2 ·yr -1) than the others. The climate productivity model has widespread overestimation and large systematic error, along with worse performances (NSEmax=0.45) and other metric indexes unsatisfactory, especially Qinghai-Tibet Plateau have relatively lower accuracy because of the available observation data is insufficient. The results from different regions also illustrated that different models for NPP estimating was suitable for variousother areas. Overall, the CASA-model is much more ideal for estimating NPP all over China in the absence of data. This study provides a comprehensive intercomparison of remote sensing, based on national NPP estimation, and anticipates future NPP simulation development at the national and global scale.
**Order of Authors:**	Jinke Sun, ph.D
	Niu HaiPeng
	Cao Dan
**Additional Information:**	
**Question**	**Response**
**Financial Disclosure**<br><br>Enter a financial disclosure statement that describes the sources of funding for the work included in this submission. Review the submission guidelines for detailed requirements. View published research articles from *PLOS ONE* for specific	The author received no specific funding for this work.

Powered by Editorial Manager® and ProduXion Manager® from Aries Systems Corporation

examples.<br><br>This statement is required for submission and **will appear in the published article** if the submission is accepted. Please make sure it is accurate.<br><br>**Unfunded studies**<br>Enter: *The author(s) received no specific funding for this work.*<br><br>**Funded studies**<br>Enter a statement with the following details:<br>• Initials of the authors who received each award<br>• Grant numbers awarded to each author<br>• The full name of each funder<br>• URL of each funder website<br>• Did the sponsors or funders play any role in the study design, data collection and analysis, decision to publish, or preparation of the manuscript?<br>• **NO** - Include this sentence at the end of your statement: *The funders had no role in study design, data collection and analysis, decision to publish, or preparation of the manuscript.*<br>• **YES** - Specify the role(s) played.<br><br>* typeset	
**Competing Interests**<br><br>Use the instructions below to enter a competing interest statement for this submission. On behalf of all authors, disclose any competing interests that could be perceived to bias this work—acknowledging all financial support and any other relevant financial or non-financial competing interests.<br><br>This statement **will appear in the published article** if the submission is accepted. Please make sure it is accurate. View published research articles from *PLOS ONE* for specific examples.	The authors have declared that no competing interests exist.

**NO authors have competing interests**<br><br>Enter: *The authors have declared that no competing interests exist.*<br><br>**Authors with competing interests**<br><br>Enter competing interest details beginning with this statement:<br><br>*I have read the journal's policy and the authors of this manuscript have the following competing interests: [insert competing interests here]*<br><br>* typeset	
**Ethics Statement**<br><br>Enter an ethics statement for this submission. This statement is required if the study involved:<br><br>• Human participants<br>• Human specimens or tissue<br>• Vertebrate animals or cephalopods<br>• Vertebrate embryos or tissues<br>• Field research<br><br>Write "N/A" if the submission does not require an ethics statement.<br><br>General guidance is provided below. Consult the submission guidelines for detailed instructions. **Make sure that all information entered here is included in the Methods section of the manuscript.**	N/A

Powered by Editorial Manager® and ProduXion Manager® from Aries Systems Corporation

**Format for specific study types**<br><br>**Human Subject Research (involving human participants and/or tissue)**<br>• Give the name of the institutional review board or ethics committee that approved the study<br>• Include the approval number and/or a statement indicating approval of this research<br>• Indicate the form of consent obtained (written/oral) or the reason that consent was not obtained (e.g. the data were analyzed anonymously)<br><br>**Animal Research (involving vertebrate animals, embryos or tissues)**<br>• Provide the name of the Institutional Animal Care and Use Committee (IACUC) or other relevant ethics board that reviewed the study protocol, and indicate whether they approved this research or granted a formal waiver of ethical approval<br>• Include an approval number if one was obtained<br>• If the study involved *non-human primates*, add *additional details* about animal welfare and steps taken to ameliorate suffering<br>• If anesthesia, euthanasia, or any kind of animal sacrifice is part of the study, include briefly which substances and/or methods were applied<br><br>**Field Research**<br><br>Include the following details if this study involves the collection of plant, animal, or other materials from a natural setting:<br>• Field permit number<br>• Name of the institution or relevant body that granted permission	
**Data Availability**<br><br>Authors are required to make all data underlying the findings described fully available, without restriction, and from the time of publication. PLOS allows rare exceptions to address legal and ethical concerns. See the PLOS Data Policy and FAQ for detailed information.	Yes - all data are fully available without restriction

Powered by Editorial Manager® and ProduXion Manager® from Aries Systems Corporation

A Data Availability Statement describing where the data can be found is required at submission. Your answers to this question constitute the Data Availability Statement and **will be published in the article**, if accepted.<br><br>**Important:** Stating 'data available on request from the author' is not sufficient. If your data are only available upon request, select 'No' for the first question and explain your exceptional situation in the text box.<br><br>Do the authors confirm that all data underlying the findings described in their manuscript are fully available without restriction?	
**Describe where the data may be found in full sentences. If you are copying our sample text, replace any instances of XXX with the appropriate details.**<br><br>• If the data are **held or will be held in a public repository**, include URLs, accession numbers or DOIs. If this information will only be available after acceptance, indicate this by ticking the box below. For example: *All XXX files are available from the XXX database (accession number(s) XXX, XXX.).*<br>• If the data are all contained **within the manuscript and/or Supporting Information files**, enter the following: *All relevant data are within the manuscript and its Supporting Information files.*<br>• If neither of these applies but you are able to provide **details of access elsewhere**, with or without limitations, please do so. For example:<br><br>*Data cannot be shared publicly because of [XXX]. Data are available from the XXX Institutional Data Access / Ethics Committee (contact via XXX) for researchers who meet the criteria for access to confidential data.*<br><br>*The data underlying the results presented in the study are available from (include the name of the third party*	http://www.resdc.cn/Login.aspx?ReturnUrl=http%3a%2f%2fwww.resdc.cn%2fUser%2fUserEdit.aspx<br>https://lpdaac.usgs.gov/data_access/

and contact information or URL).<br>• This text is appropriate if the data are owned by a third party and authors do not have permission to share the data.<br><br>* typeset	
Additional data availability information:	

Manuscript

Click here to access/download;Manuscript;Evaluation of NPP using three models compared over China .docx

Evaluation of NPP using three models compared with MODIS-NPP data over China

Jinke Sun[1], Dan Cao[2], Haipeng Niu[1]

[1]School of Surveying and Land Information Engineering, Henan Polytechnic University, Jiaozuo, Henan, China
[2]Key Laboratory of Digital Earth Science, Institute of Remote Sensing and Digital Earth, Chinese Academy of Sciences, Beijing, China;

Correspondence author: Haipeng Niu (Email: niuhaipeng@126.com)

ABSTRACT Estimating net primary productivity (NPP) is crucial in global climate change research and carbon cycling. However, there are many uncertainties different NPP modeling results and the process ofNPP is challenging to model on the absence of data. In this study, we ~~only~~ used meteorological data as input, the estimated NPP using climate productivity model, synthetic model and CASA model were compared with MOD17A3-NPP as the reference data over China during the past 16 years (2000-2015). Then the statistics evaluation metrics (Relative Bias (RB), Pearson linear Correlation Coefficient (CC), Root Mean Square Error (RMSE), and Nash-Sutcliffe efficiency coefficient (NSE)) for three models with the reference data were calculated. The correlation analysis implied that the CASA-model performed better than the other two models in terms of RB, RMSE, NSE and CC whether on the national or the regional scale. It has a higher CC with 0.5081 and a smaller RMSE with 111.96 g $C·m^{-2}·yr^{-1}$ in the whole country. The synthetic model and CASA-model has the same advantages at some regions, and the artificial model has lower RMSE in Southern China (86.35 g $C·m^{-2}·yr^{-1}$), Xinjiang (85.53 g $C·m^{-2}·yr^{-1}$) and Qinghai-Tibet Plateau (93.22 g $C·m^{-2}·yr^{-1}$) than the others. The climate productivity model has widespread overestimation and large systematic error, along with worse performances (NSEmax=0.45) and other metric indexes unsatisfactory, especially Qinghai-Tibet Plateau have relatively lower accuracy because of the available observation data is insufficient. The results from different regions also illustrated that different models for NPP estimating was suitable for variousother areas. Overall, the CASA-model is much more ideal for estimating NPP all over China in the absence of data. This study provides a comprehensive intercomparison of remote sensing, based on national NPP estimation, and anticipates future NPP simulation development at the national and global scale.

Keywords : NPP estimation, climate productivity model, Synthetic model, CASA-model, MOD17A3, statistics evaluation

I. Introduction

NPP is the amount of organic matter produced by photosynthesis minus autotrophic respiration, which is defined as the net amount of organic matter fixed by plants through photosynthesis. It represents the net carbon flow from the atmosphere to the terrestrial ecosystems and is affected by many factors, such as climate, soil, nutrients and CO_2 [1]–[3]. Generally, assuming that vegetation can make full use of the climate resources, such as light, heat ,and water when other factors are in the optimum state, which can obtain the maximum biological or agricultural yield per unit area of land is called climatic production potential[4], [5]. As one of the critical indicators of ecosystem function, NPP reflects the growth of vegetation and the health status of ecosystems [6] as well as changes in climate and ecosystem function change [7], and it is useful in modeling researches of regional and global carbon and water cycles. Therefore, a better understanding of NPP estimates is essential for predicting the future carbon flux of terrestrial ecosystems in the context of global warming.

Traditionally, NPP estimations were based on field surveys and fixed-point observations. However, these methods are not feasible on a large scale because of its low efficiency, high cost, and inability to obtain complete spatial coverage and continuous long-term time series [8], [9]. With the development of satellite remote sensing technology, we can find a powerful way to retrieve NPP's spatial distribution inland vegetation. MOD17A3-NPP, as one existing large scale product acquired by remote sensing, has 1 km resolution and complete applications in different ecosystems and is often recognized as better validation data for the large-scale spatial coverage [10] – [12]. Additionally, more and more scholars estimated vegetation NPP using remote sensing models in different regions of the world as well as analyzed its temporal and spatial distribution features from different angles [7],

[13]－[16]. Generally, there are large uncertainties due to the lack of data in the large-scale NPP calculation. [17]–[19]. Therefore, models that require fewer data have a more significant advantage over large-scale for NPP simulation, such as climate productivity models (e.g. Miami model (Lieth, 1975), Thornthwaite Memorial model (Lieth, 1975)), synthetic model [20], [21], light to use efficiency models (e.g. CASA modles [22]) and so on. Climate productivity models are always used to estimate potential productivity as the maximum regional productivity [1].In previous studies, many researchers also chose the climate productivity model [23] and synthetic model [20], [21] to estimate the potential productivity [1]. The artificial model is established mainly based on the measured biomass data, which is from 125 stations connected with natural mature and 23 stations related to natural vegetation NPP in China such as grassland, and desert [6]. Meanwhile, there is a notable advantage for estimating actual productivity based on the CASA model as a light use efficiency model [24]–[27] and the current version of the CASA model take into account land-cover change[28]. However, there are many differences and uncertainties during these models. Systematic validation of those models is rare in large-scale regions, which may influence our understanding of the ecosystem's carbon balance and assess vegetation response to climate. Moreover, most studies have focused on a single NPP estimated model and the results of various methods used to have considerable differences in uncertainty. Evaluating the reliability of results acquired by different models and assessing the differences are still open questions.

In this study, we only used meteorological data as input data, the estimated NPP using climate productivity model (Thornthwaite Memorial model), synthetic model and CASA model were compared with MOD17A3-NPP as the reference data over China during the past 16 years (2000-2015). Then the statistics evaluation metrics (Relative Bias (RB), Pearson linear Correlation Coefficient (CC), Root Mean Square Error (RMSE), and Nash-Sutcliffe efficiency coefficient (NSE)) for three models with the reference data were calculated. The study results will provide baseline data for large-scale and long-time series NPP evaluation and help the policymakers understand the current situation of NPP spatial distribution in China and develop environmental policies related to crop production.

II. Materials and methods

A. Study area

China, the third-largest ~~county~~ globally, is located in the East of Asia on the western shore of the Pacific Ocean.

China spans tropical, subtropical, warm temperate, middle temperate and cold temperate climate zones from south to north. Mild monsoon climate, subtropical monsoon climate, tropical monsoon climate, tropical rainforest climate, temperate continental climate and plateau mountain climate dominate different regions in China, and the diversity of climate types results in various vegetation types[29]. To clearly to distinguish NPP in China under other climatic and topographic conditions, we divide China into seven major regions and each of which may contain one or more administrative areas. The seven significant regions include：Ⅰ) the Xinjiang (XJ) region, which has arid and semi-arid climate characteristics. Ⅱ) Qinghai－Tibet Plateau (TP) which has an average elevation of about 4500m. Ⅲ) Northwestern China (NW) bounded by the 400 mm annual precipitation isohyet. Ⅳ) Northeastern China (NE) is located in the north of the Yan mountains. Ⅴ) Northern China (NC) is located in the north of the Qinling Mountains–Huai River line and the vegetation patterns in this region are characterized by a mosaic of agricultural vegetation. VI) Yunnan–Guizhou Plateau in southwestern China (SW)is bounded by the Ta-pa Mountains and Wulingshan Mountains to the north and east. VII) and Southern China (SC) south of the Nanling Mountains and Southeast of the Wuyi Mountains. Southern China (SC) south of the Nanling Mountains and Southeast of the Wuyi Mountains. The same China divisions are from [30], [31] .These subregions abbreviations are labeled in Fig1a and will be used herein.

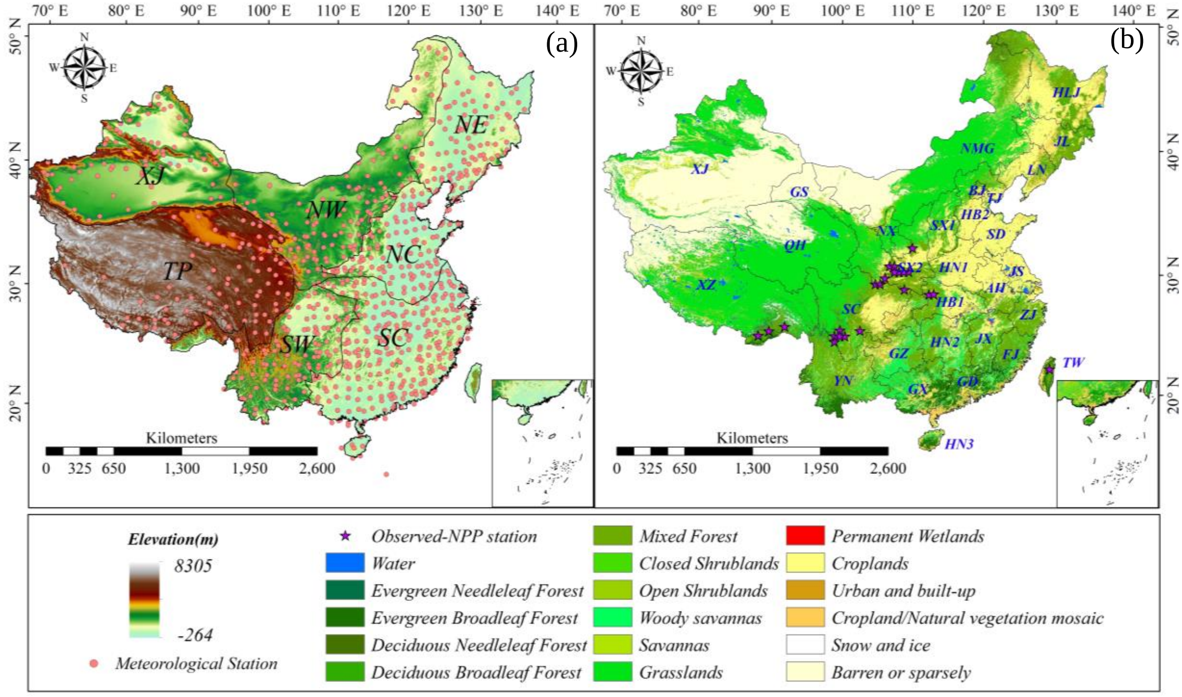

Fig. 1 (a) Different regions of the study area according to the elevation and annual precipitation. The pink circles are 839 meteorological stations. (b) Land use of China. The 23 stars represent the observed-NPP stations from published papers, and these stations located in the temperate forest are mainly used to verify the potential productivity

B. Data and processing

1) REMOTE SENSING DATA

a. MODIS NPP data

The MOD17A3's global NPP data for 2000-2015 was used as the reference data in this study. These data were obtained from NASA's website (https://lpdaac.usgs.gov/data_access/) with 1 km spatial resolution. It contains total primary productivity (GPP), net primary productivity (NPP) and net direct quality control (NP_QC). In this study, 21 images in China were selected. The NPP data from 2000 to 2015 were converted into NPP data with unit g $C\cdot m^{-2}\cdot yr^{-1}$, and the scale coefficient was 0.1(Fig.2). Studies found that MOD17A3 NPP dataset has outstanding agreements with the observations at the global or country scale[32]–[34].

b. MODIS NDVI data

MODIS normalized difference vegetation indexes(NDVI) product with a 250m/16-day spatiotemporal resolution between 2000 and 2015 was used in this study. These data were from the MODIS product MOD13A1. The maximum-value composite method generated the monthly NDVI data and then were reprojected to the Albers equal-area projection. The NDVI data were then used to drive CASA-model as input data for NPP estimation.

2) METEOROLOGICAL DATA

This study used meteorological data such as daily temperature, daily precipitation, and solar radiation from 2000 to 2015. All data is provided by the China Meteorological Administration (http://cdc.nmic.cn/home.do), obtained from the 839 meteorological stations (130 solar radiation stations) in the whole country. Meteorological data driving the CASA model include monthly precipitation, monthly mean temperature, and monthly solar radiation. The data supplied to drive the Thornthwaite Memorial and Zhou model are only yearly temperatures and precipitation datasets. These data were interpolated using ANUSPLIN (version 4.2) to create regular monthly data or yearly data layers with the same spatial resolution as MOD17A3-NPP.

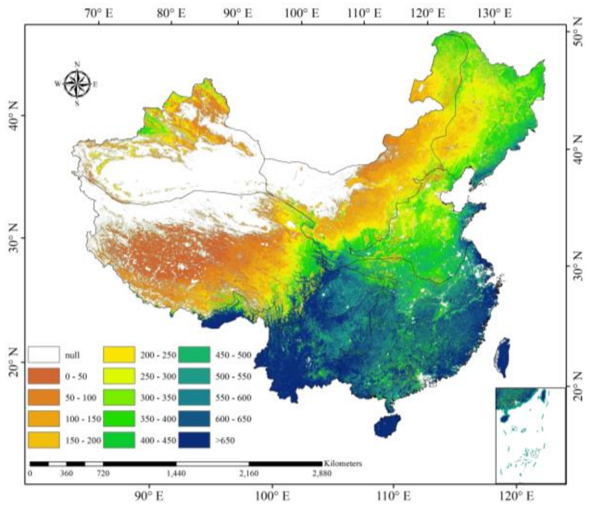

Fig. 2 The spatial distribution of multi-year MOD17A3-NPP average from 2000 to 2015

3) LAND USE DATA AND OTHERS

Land use maps were from the MODIS product of MCD12Q1 and obtained by NASA (https://lpdaac.usgs.gov/data_access/). The data are classified into 14 categories according to IGBP global vegetation classification scheme, including the Evergreen Needleleaf Forest, Evergreen Broadleaf Forest, Deciduous Needleleaf Forest, Deciduous Broadleaf Forest, Mixed Forest, Closed Shrublands, Open Shrublands Woody Savannas, Savannas Grasslands, Permanent Wetlands, Croplands, Urban and built-up, Cropland/natural vegetation mosaic, Snow and Ice, and Barren or Sparsely land (Fig.1b).

DEM data with 1km resolution was from the Resource and Environment Data Cloud Platform, which was mainly used for This study used meteorological data such as meteorological data interpolation as affecting factors of temperature and precipitation.

4) IN-SITU SURVEY DATA

In this study, the verification NPP data were derived from the study of [35], with the Global Primary Productivity Initiative (https://daac.ornl.gov/). These data come from the National Forest Resources Inventory conducted by the Chinese Forestry Department during the period 1989-1993. Besides, we also used in-situ survey datasets from published kinds of literature with well-documented field sites [1], [36]–[38]. These data provided some valuable information such as site names, latitude, longitude, elevation, biomass and NPP estimations for most of the plant components. Mostly, the data from Global Primary Productivity Initiative covered representative sample points of different vegetation types, and the data are widely used in global model parameterization and result verification. All data included different vegetation types and total NPP data of China's administrative districts. Finally, 23 observed-NPP stations (Fig.1b) and unlimited amounts of every administrative community were collected as NPP validation data nationwide.

C. Methods

1) CLIMATE PRODUCTIVITY MODEL FOR ESTIMATING NPP

Thornthwaite Memorial[39], as one climate productivitymodel, established a statistical relationship between Net Primary Productivity (NPP) and evapotranspiration (ET) based on the relationship between evapotranspiration, temperature, precipitation, and vegetation. On this basis, Lieth (1975) proposed the Thornthwaite Memorial model in 1974 based on the vegetation NPP in 50 different locations on 5 continents. The climate factors considered in this model are relatively simple and can better reflect the key factors affecting plant growth and development, such as temperature, precipitation, and evapotranspiration. The calculation formula is ．

$$NPP_T = 3000 \times [-e^{-0.0009695(v-20)}] \tag{1}$$

$$v = \frac{1.05r}{\sqrt{1 + (1 + \frac{1.05r}{L})^2}} \tag{2}$$

$$L = 300 + 25T + 0.05T^3 \tag{3}$$

where, v: Annual average actual evapotranspiration (mm), r: Annual average precipitation (mm), L: the maximum annual evapotranspiration (mm), expressed as a function of the average annual temperature T (℃). NPP_T is the climate productive potential of vegetation determined by evapotranspiration that was calculated

in units of g DW/m^2/yr. This was implemented by applying a conversion factor of 0.475 [28] in China from dry matter (DW) to carbon content (g C·m^{-2}·yr^{-1}).

2) SYNTHETIC MODEL FOR ESTIMATING NPP

According to the physiological and ecological characteristics of plants and the regional evapotranspiration model based on the energy balance equation and the water balance equation, Zhou (1995) and Zhang(1989) established the NPP model of natural vegetation combining the physiological and ecological characteristics of plants and the relationship between water and heat balance. The calculation formula [1] is as follows:

$$NPP = 100 \times RDI \frac{rR_n(r^2 + {R_n}^2 + rR_n)}{(R_n + r)({R_n}^2 + r^2)} exp(-\sqrt{9.87 + 6.25RDI}) \quad (4)$$

$$R_n = RDI \times r \times L \quad (5)$$

$$RDI = (0.629 + 0.237PER - 0.00313PER^2)^2 \quad (6)$$

$$PER = \frac{PET}{r} = \frac{BT \times 58.93}{r} \quad (7)$$

$$BT = \frac{\sum t_d}{365} \quad (8)$$

$$L = 597 - 0.6T_m \quad (9)$$

NPP was calculated in units of g DW·m^{-2}·yr^{-1} and was implemented by applying a conversion factor of 0.475 in China from dry matter (DW) to carbon content (g C·m^{-2}·yr^{-1}). *r* is average annual precipitation, R_n is annual net radiation, *RDI* is radiant dryness, *L* is the latent heat of evaporation, *PET* is potential evapotranspiration, *PER* is probably evapotranspiration rate. Biological temperature (BT) is the average temperature experienced during plant growth, generally between 0 and 30℃. Mean daily temperature (t_d) and mean monthly temperature (T_m) takes0℃ when it lowers than 0℃ and can be calculated at 30℃ when the temperature is higher than 30℃.

3) CASA-MODEL FOR ESTIMATING NPP

The Carnegie-Ames-Stanford Approach (CASA) models[13], [22], a satellite-based photosynthetic utilization models, is widely used to calculate the NPP. The CASA model requires the following parameters, such as temperature, rainfall, solar radiation, NDVI, etc. The model can be calculated by APAR (Absorbed Photosynthetic Active Radiation) times the light energy conversion rate ε. The calculation expression is as follows:

$$\mathrm{NPP}(x,t) = \mathrm{APAR}(x,t) \times \varepsilon\,(x,t) \quad (10)$$

$$\mathrm{APAR}(x,t) = \mathrm{FAPAR}(x,t) \times \mathrm{PAR}(x,t) = \mathrm{SOL}(x,t) \times \mathrm{FAPAR}(x,t) \times 0.5 \quad (11)$$

where, NPP(x,t) represents NPP fixed by vegetation at a grid cell x in month t. APAR(x,t) represents photosynthetically active radiation absorbed by green vegetation. ε(x,t) represents actual the utilization of light energy. SOL(x,t) represents the total solar radiation of pixel x in month t. PAR is the incident photosynthetically active radiation (M J m-2) per month. FPAR(x,t) is the fraction of PAR absorbed by the vegetation canopy. Constant 0.5 represents the solar effective radiation ratio that vegetation can utilize, namely, the ratio of PAR divided by SOL. The detail of the CASA-model can be found in the study of Potter (1993) and Zhu (2007).

4) VALIDATION AND STATISTICAL EVALUATION METRICS

In this study, we validated the estimated NPP by comparing it with measured data. These data were collected from Luo's investigation data[35] and other published literature. We are also the focus on the evaluation of different results from three models (Thornthwaite Memorial model, Synthetic model and CASA-model) in this study. The average result of MOD17A3-NPP was used as the reference simulation compared with the NPP result using three models. A series of traditional error indexes, which include Bias, Relative Bias(RB), Pearson linear Correlation Coefficient (CC), Root Mean Square Error (RMSE), and Nash-Sutcliffe efficiency coefficient (NSE), is calculated at a pixel in this study. RB, NSE ,and CC are dimensionless, and RMSE is in gC/m^2. RB, when multiplied by 100, denotes the degree of overestimation or underestimation in percentage. The definition of RB, CC, and RMSE can be found in [30], [31], [40], and NSE that is generally used to verify the quality of the hydrological model simulation results are defined as follows:

$$NSE = 1 - \frac{\sum_{t=1}^{T}\left(Q_0^t - Q_m^t\right)^2}{\sum_{t=1}^{T}\left(Q_0^t - \bar{Q}_0\right)^2} \quad (12)$$

Q_0 : the result of the reference model;Q_m : the simulated value of the comparison model;

$\bar{Q}_0$s: the annual mean of the model data;

t :the year;

The value range of *NSE* is (-∞, 1), and the closer the value is to 1, the higher the similarity between the comparison models and the reference models are. The closer the value is to 0, the closer the simulation result of the comparison model is to the result of the reference model, that is, the more reliable the overall result is., but the process simulation error of that is large. When the value of *NSE* is far less than 0, it indicates that the model is not credible [40].

III. Results and discussion

A. Validation of estimated NPP

In this study, we validated the estimated NPP at the provincial scale (Fig.3a and 3b) and the station scale (Fig.3c and 3d), respectively. The results showed that the simulated average annual NPP in each province from 2000 to 2015 using the three models (Thornthwaite Memorial model, Synthetic model and CASA-model) in this study are highly correlated with the result of Luo during 1989 to 1993 (Fig.3b). R^2 was 0.5188, 0.6653 and 0.692, respectively, and the corresponding RMSE was 1.67 $t{\cdot}ha^{-1}{\cdot}yr^{-1}$, 0.6653 $t{\cdot}ha^{-1}{\cdot}yr^{-1}$ and 0.692 $t{\cdot}ha^{-1}{\cdot}yr^{-1}$, respectively. However, the Thornthwaite Memorial model and Synthetic model's annual average values are higher whereas the CASA-model are lower than those of Luo (Fig.3a) at the provincial scale. It may be caused by various uncertainties such as time inconsistency and the former two models are used to estimated potential productivity. In addition, we also compared MODIS NPP products with the observed data. The results (R^2=0.81) showed that MODIS NPP products can meet the needs of long time series NPP variation. . Fig. 3c and 3dalso illustrated CASA model was better than the other two models at the station scale and the results of CASA model were more closer MODIS NPP products, which is likely due to CASA-model and MOD17A3 products both belong to the light use efficiency(LUE) model. The validation results provide a basis for comparing the subsequent results on grid-scale with reference data. Besides, the total NPP of China land simulated by the Thornthwaite Memorial model, Synthetic model and CASA-model were 4.03Pg C (1Pg C=10^{15}g C), 2.47 Pg C and 3.58 Pg C, respectively. This is within the reported values of 1.95-6.13 Pg C[28], [41], [42]. We also compared the NPP calculated from the three models with other simulation results (Table 1). This indicated the reliability of our results.

Table 1 Comparison of annual average NPP between this study and other studies

Reference	Studied period	Studied area	model	Precision
This study	2000-2015	China	TW model	R^2 = 0.61
			ZGS model	R^2 = 0.76
			CASA model	R^2 = 0.78
(Zhou et al., 2013)	2001-2010	Heihe River Basin	TW model	None
[1]	2000-2015	The Loess Plateau	ZGS model	R^2 = 0.734, P <0.01
[1]	2000-2015	The Loess Plateau	CASA model	R^2 = 0.817, P <0.01
[42]	2001-2010	China	CASA model	r=0.733,P<0.001
[43]	2000-2014	The Ili River Valley	CASA model	R^2 = 0.65, P <0.01,
(Zhu et al., 2007)	1982-2000	China	CASA model	RB=4.5%

Note: For charting and table simplification, TW represents the climate productivity (Thornthwaite Memorial) model. ZGS represents Synthetic model proposed by Guangsheng Zhou, the same below. The R2 at station scale used in this table.

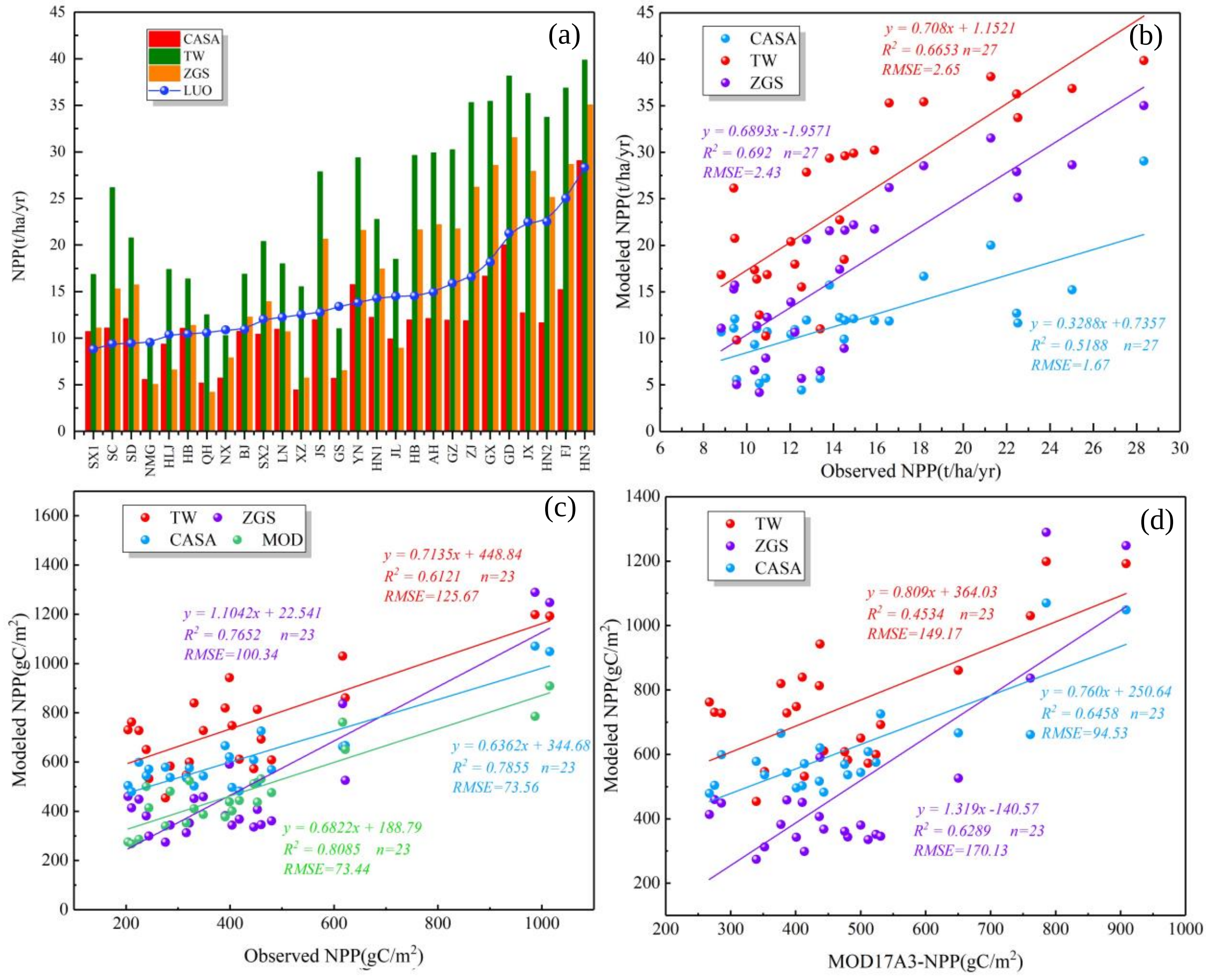

Fig.3 Validation of NPP (a). The range of NPP for three models and LUO's measured data in 27 provincial administrative regions (b). Validation of NPP for three models and LUO's measured data in 27 provincial administrative regions(R2 and RMSE) (c). Validation of NPP for three models and reference data MOD17A3 in 23 observed stations (R2 and RMSE) (d). Validation of NPP for three models and reference data MOD17A3 in 23 observed stations (R2 and RMSE).

B. Spatial distribution of NPP

Fig.4 showed that the spatial distribution of estimated NPP was consistent with those of the reference data. The general trend of NPP distribution in China is higher in the Southeast and lower are the northwest. The spatial distribution of productivity varies from year after year due to different climatic factors, topographic factors, phenological characteristics , and vegetation types. As shown in Fig.4, there are pronounced regional differences in every model, offering a gradually decreasing trend from Southeast to northwest. In southern China, the evergreen broad-leaved forest is widely distributed and rich in resources. The annual average NPP is higher than 600 g $C\cdot m^{-2}\cdot yr^{-1}$. Rich precipitation groundwater and a warm climate are more conducive to the growth of vegetation [9]. However, the average annual NPP is lower than 200 g C·m-2·yr-1 in the northwest due to poor soil, low temperatures and low rainfall [28]. Overall, the maximum value of NPP during 2000-2005, occurred in the southwest, southern China and Taiwan. The values between 600 and 700 g $C\cdot m^{-2}\cdot yr^{-1}$ were located in the south of the lower reaches of the Yangtze River, east of Yunnan-Guizhou Plateau and north of Nanling Mountains with annual NPP. Annual NPP between 400 and 600 g $C\cdot m^{-2}\cdot yr^{-1}$ were located in Daxing'an Mountains, Xiaoxing'an Mountains, east of Taihang Mountains, middle reaches of the Yangtze River Basin, most area of Sichuan, southeastern Tibet, Tianshan Mountains in Xinjiang and Altai Mountains with. The low-value sites mainly distribute in Inner Mongolia, Xinjiang, Qinghai-Tibet Plateau, and parts of Shanxi, Gansu, Ningxia and Shanxi provinces with annual NPP less than 200 g $C\cdot m^{-2}\cdot yr^{-1}$.

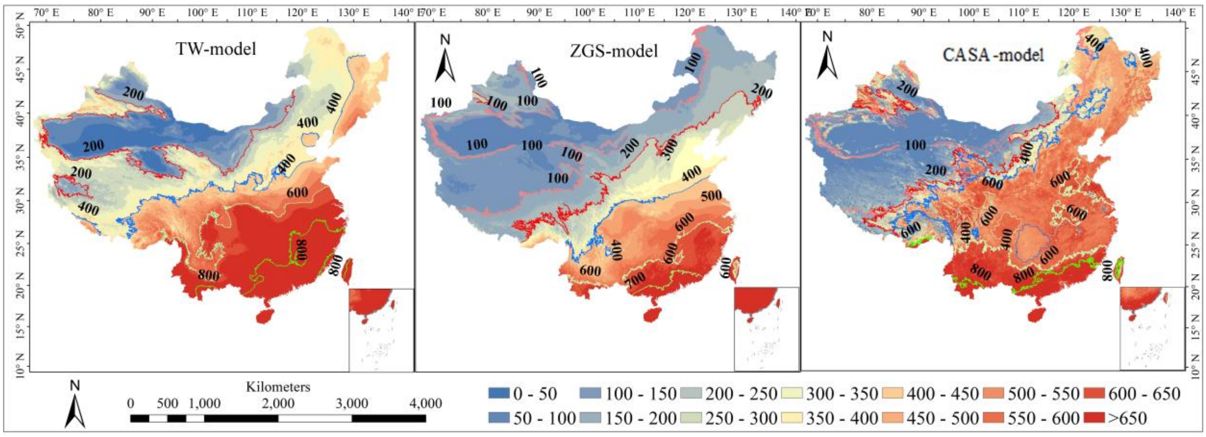

Fig.4 The spatial distribution of NPP from three models

However, there was also a discrepancy in different models. Spatial patterns of 16 years average NPP over China depicted by CASA-model, on the other hand, agree with the reference MOD17A3. The difference is that the Thornthwaite Memorial model and Synthetic models simulation results showed obvious banded distribution from northwest to Southeast, and the NPP contour increased steadily from northwest to Southeast, which was relatively smooth. The simulation results of the CASA model showed a larger zigzag shape, especially in the southern region, the simulation results had no obvious regional regularity. Additionally, the Thornthwaite Memorial model, a climate productivity potential modelas obviously overestimated compared with the other two models and noted that the Thornthwaite Memorial model results reveal very high maxima at individual pixels especially in southern China. The Synthetic model's result showed close spatial patterns with the Thornthwaite Memorial model than those of the CASA-model in the contour line, and the contour lines of the Synthetic model showed obvious hysteresis phenomenon in the northwest-southeast direction. Synthetic model and CASA-model consider the influence of evapotranspiration on the model simulation results in the parameter input process. Still, the original input data of onto Synthetic model areis the same as the Thornthwaite Memorial model. At the same time, CASA-model takes into account the effects of radiation and land to use category on the results. Therefore, the results of CASA-model are closer to actual productivity.

Table 2 showed the relative bias (RB) between the annual average NPP of the three models and the reference data. Interestingly, the overall relative error is the Synthetic model (18.54%) less than CASA-model (21.34%) and the Thornthwaite Memorial model (30.34%). It indicated that the Synthetic model was underestimated whereas CASA-model and Thornthwaite Memorial model overestimated from the country scale. The relative precision decreased from 81.47% in the Synthetic model to 78.66% in CASA-model and 69.66% in the Thornthwaite Memorial model. Regionally, the Thornthwaite Memorial model results were overestimated in most regions, and the relative deviation are very high expectations for the region of SW (8.08%), which was only higher than that of CASA-model (6.86%) simulation in this region. The results of CASA-model were also largely overestimated compared with the reference data in most regions, especially in XJ, TP , NW, arid areas of Northwest China and plateau areas. The annual average estimation NPP of Synthetic models on SC, NC, TP, and NW were in good agreement with the reference data, and the relative deviation in other regions were more than 30%. . All in all, the Synthetic model and CASA-model had the same advantage in overall simulation accuracy, which can be used in the absence of data.

Table 2RB for the results of three models comparable with reference data (Note: TW represents Thornthwaite Memorial model)

	MOD_NPP (g C/m^2)	TW_NPP (g C/m^2)	TW_RB (%)	ZGS_NPP (g C/m^2)	ZGS_RB (%)	CASA-NPP (g C/m^2)	CASA-RB (%)
SC	617.34	771.00	24.89	596.07	-3.45	682.98	10.63
NE	318.24	381.16	19.77	177.31	-44.28	357.95	12.47
NC	351.80	470.72	33.80	353.65	0.53	461.89	31.29
XJ	58.01	136.84	135.89	76.52	31.91	119.71	106.36
TP	109.24	334.50	206.21	114.17	4.51	223.48	104.58
SW	578.08	531.38	-8.08	338.64	-41.42	538.45	-6.86
NW	167.79	242.66	44.62	136.12	-18.87	285.67	70.25
ALL	314.36	409.75	30.34	256.07	18.54	395.73	21.34

C. Subregional statistics evaluation of NPP

NSE (Fig 5) for the simulation of three models showed the results from CASA-model was more consistent with the reference data. This was likely due to the models of two results were belonged to the LUE model. However, the NSE of CASA-model was close to 0 or negative infinite values in a few areas, such as the Qinghai-Tibet Plateau, which indicated that there was inadaptable in CASA-model in this area. This is likely because the meteorological stations are few and unevenly distributed, resulting in the error of interpolation results. The maximum value of NSE from the Synthetic model can reach 0.73 in the northeast and south of NE and NW. And it is greater than 0.5 in most areas of SW, the eastern part of TP and the edge of Xinjiang. Some researches indicated that the performance levels were defined as follows: NSE > 0.65 = excellent, 0.65 - 0.5 = very good, 0.5 - 0.2 = good, and < 0.2 = poor [40], [45]. NSE in most areas of central NC and some areas of southern NC was near 0.5, which indicated that Synthetic model had certain applicability in these areas. However, the simulation results of some areas such as the central and western parts of TP, the edge parts of Xinjiang and the parts of NC and SC border appear near 0. Compared with the simulation results of the two models mentioned above, the NSE simulation results of the Thornthwaite Memorial model were less than 0.5, it illustrated that there were great differences between the simulation result of the Thornthwaite Memorial model and MOD17A3.

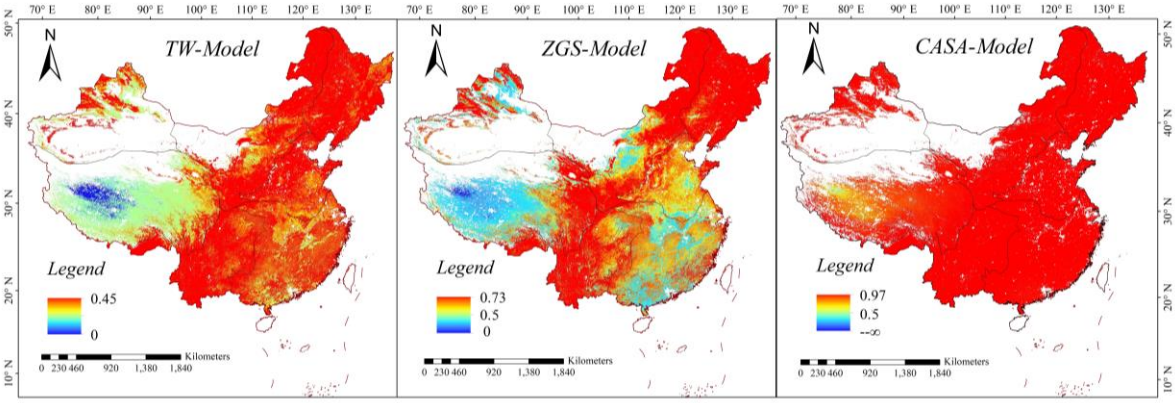

Fig.5 The spatial distribution of NSE for three models compared with reference dataset

The average RMSE (Fig 6) of the three models was less than 200 g $C \cdot m^{-2} \cdot yr^{-1}$, within the allowable range of their respective errors in the whole country. Table 3 indicated that CASA-model performed well noticeably in NE, XJ, SW and NW. The average of RMSE increased from 111.96 g $C \cdot m^{-2} \cdot yr^{-1}$ in CASA-model to 133.14 g $C \cdot m^{-2} \cdot yr^{-1}$ in Synthetic model and 172.46 g $C \cdot m^{-2} \cdot yr^{-1}$ in Thornthwaite Memorial model. It showed the result from CASA-model was consistent with the reference data. In terms of spatial distribution, the RMSE of CASA-model was 0-150 g $C \cdot m^{-2} \cdot yr^{-1}$ in the whole region, especially in Inner Mongolia Autonomous Region, XJ, most areas of NE and Shandong Peninsula where RMSE was less than 50 g $C \cdot m^{-2} \cdot yr^{-1}$. RMSE was more than 200 g $C \cdot m^{-2} \cdot yr^{-1}$ in the central of NC and TP. The larger RMSE occurs in southern SC, Southern SW and southern Tibet. However, the Synthetic model also had preponderance in most areas of NC, XJ and TP, and the range of RMSE was between 150-250 g $C \cdot m^{-2} \cdot yr^{-1}$ in the northeastern margin area. The differences between simulated NPP and reference data were more considerable in most areas of SC and the junction area between TP and SC where RMSE was more significant than 400 g $C \cdot m^{-2} \cdot yr^{-1}$. Compared with the above two results, the RMSE of Thornthwaite Memorial model was higher in the whole country. In summary, the error of CASA-model and MOD17A3 reference data is the smallest, followed by Synthetic model, and the worst is Thornthwaite Memorial model.

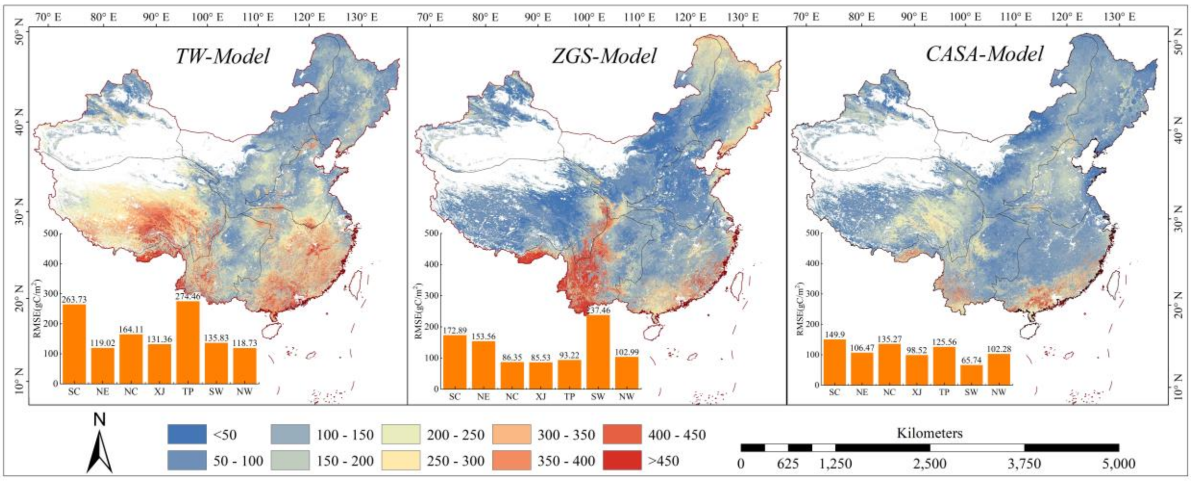

Fig.6 The spatial distribution of RMSE for three models compared with reference dataset

Table 3

RMSE of three models comparable with reference data

	TW-RMSE ($g\ C/m^2$)	ZGS-RMSE ($g\ C/m^2$)	CASA-RMSE ($g\ C/m^2$)
SC	263.73	172.89	149.9
NE	119.02	153.56	106.47
NC	164.11	86.35	135.27
XJ	131.36	85.53	98.52
TP	274.46	93.22	125.56
SW	135.83	237.46	65.74
NW	118.73	102.99	102.28
Average	172.46	133.14	111.96

CC in the CASA-model, Thornthwaite Memorial model and Synthetic model were not good consistent with those resulting in the reference data. The average CC in the CASA-model was 0.5081 higher than CC in Synthetic model and Thornthwaite Memorial model (Fig.7) in the whole country. However, the average values showed large regional differences. The CC of three models in NW and NE regions was higher than the other regions. It illustrated that estimated NPP in these regions using CASA-model, Synthetic model and Thornthwaite Memorial model were well consistent with MOD17A3-NPP. However, the SC region captured the worst result in three models, as well as SW、TP、XJ and NC regions in the Thornthwaite Memorial model and Synthetic model except for CASA-model, which indicated that the models had a discrepancy in these areas. Fig.8 showed the differences of spatial distribution in three models were in different regions. In most areas of NE, NW, central NC and SW, the simulation results were better with those of reference data where CC can reach more than 0.8, showing a positive correlation. However, there was a negative correlation with CC between -1 and -0.8 in the southern part of TP and the border area between TP and SW, the eastern part of NE, the XJ boundary, Shandong Peninsula and the southern coastal area of SC. The overall performance of CASA-model is a positive correlation with MOD17A3 data. However, there was a negative correlation that was lower than the above two models in most NE areas, most areas of TP and SC.

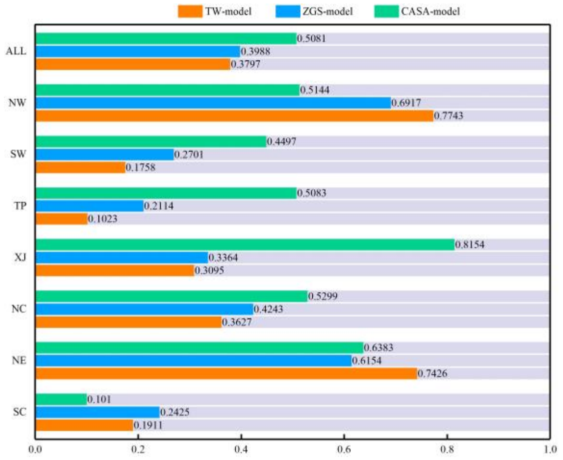

Fig.7 CC for three models compared with reference dataset

D. Uncertainties

The formation process of vegetation NPP is affected by many factors related not only to various physiological and ecological factors of the vegetation itself but also to many complicated environments. However, the three models estimation process and the MOD17A3-NPP used in this study take fewer factors into account. The three models were all relatively simple, especially the MOD17A3-NPP was known to underestimate NPP in areas with high productivity, and overestimate NPP in low productivity areas [33]. There are still large uncertainties because the real situation was not entirely the same with the simulation. Due to the different physiological mechanisms of various ecosystems, such as forest ecosystem, grassland ecosystem and farmland ecosystem. Many scholars have proved there are obvious spatial-temporal variations between different vegetation types [7], [28], [41], [46], [47]. Although the validation data used in this study adopt the multi-year average of eliminating systemic errors, the inconsistency of time intervals will inevitably lead to errors. Additionally, the limitation of spatial resolution of remote sensing image data, a large number of mixed pixels are generated in the image, which affects NPP's simulation results. Moreover, as for the multi-year NPP average, the uncertainty in simulated NPP also resulted from climate input data such as the differences in temperature, precipitation, topography and other aspects at the station scale through interpolated tools. Nonetheless, the accuracy of extraction results and the difference between the three models are acceptable in the absence of data. This study provides new insight for large-scale and long-time series NPP evaluation and helps to understand the difference of various models.

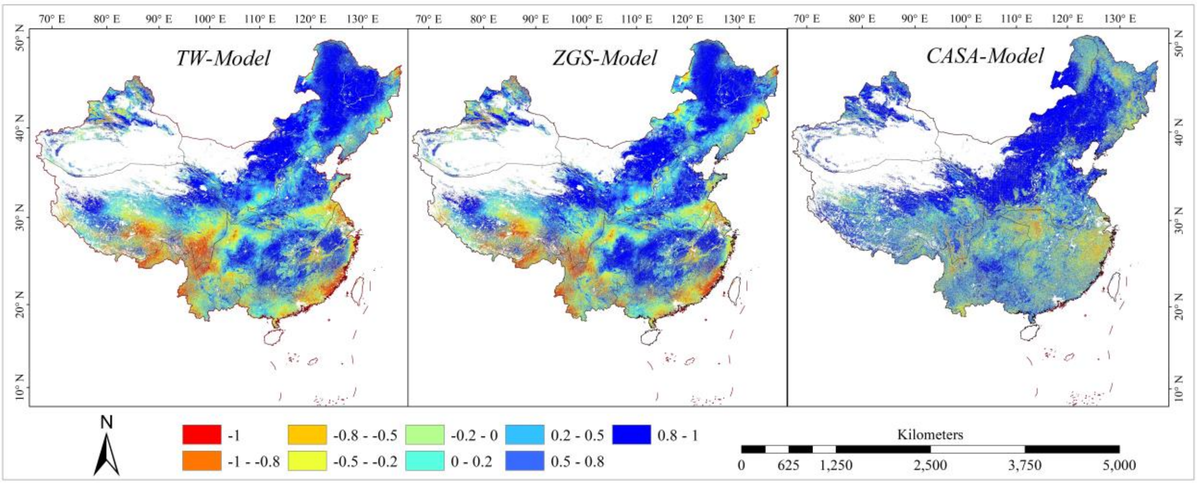

Fig.8 The spatial distribution of CC for three models compared with reference dataset

IV. Conclusions

In this study, NPP simulations of the climate productivity model, Synthetic model and CASA-model were compared within-site measured and MOD17A3-NPP as the reference data, and the multi-year NPP average from the three models were estimated in China during2000-2015. In addition, the statistics evaluation metrics (RB, RMSE, NSE, CC) for three models with the reference data were calculated. The results showed that NPP simulations of the above three models were all within the reported values comparable to other's results from 2000 to 2015. The comparable results happened in the three models and reference data also illustrated that there are

great differences. On the one hand, spatial patterns of NPP decided by the Thornthwaite Memorial model, Synthetic model and CASA-model disagree with the reference MOD17A3-NPP. The results overestimated using the Thornthwaite Memorial model as a climate productivity model, mainly used to estimate potential productivity. The Synthetic model and CASA-model had same advantages from a regional perspective, and Synthetic model had lower RMSE in the region of SC (86.35 g C·m^{-2}·yr^{-1}), XJ (85.53 g C·m^{-2}·yr^{-1}) and TP (93.22 g C·m^{-2}·yr^{-1}) than others. On the other hand, CASA-model had a great model goodness degree, correlation analysis and performed better in estimating actual NPP amounts than the other two models according to statistics evaluation metrics such as RB, RMSE, NSE and CC in the whole country. In addition, the difference between the three models occurred mainly in different ecosystems. The three models revealed very high maxima at the individual pixels, especially in southeast China where there are a mixed forest, urban and built-up. In summary, the CASA-model agrees well with the reference data which can be used to estimated NPP in the absence of data. Importantly, these results can provide powerful help for researchers to select the appropriate NPP evaluation model.

V. Acknowledgments

The authors gratefully acknowledge the financial supports by the Innovative research team of Henan Polytechnic University (Grant No. T2018-4).

REFERENCES

[1] C. Gang, W. Zhao, T. Zhao, Y. Zhang, X. Gao, and Z. Wen, “The impacts of land conversion and management measures on the grassland net primary productivity over the Loess Plateau, Northern China,” *Sci. Total Environ.*, vol. 645, pp. 827–836, 2018.

[2] N. J., “Estimating net primary productivity of grassland from field biomass measurements in temperate northern China,” *Plant Ecol.*, vol. 174, pp. 217–234, 2004.

[3] H. Tian *et al.*, “Model estimates of net primary productivity, evapotranspiration, and water use efficiency in the terrestrial ecosystems of the southern United States during 1895–2007,” *For. Ecol. Manag.*, vol. 259, no. 7, pp. 1311–1327, 2010.

[4] J. M. Liu and SHEN, “A dynamic division scheme of agro-climatic production potential for winter wheat in Beijing,Tianjin and Hebei province,” *J. Meteorol. Environ.*, 2010.

[5] Z. Tian *et al.*, “Analysis of plant climatic production potential and it’s restricting factors of 1 km2 grid of Beijing mountain area,” *J. China Agric. Univ.*, 2004.

[6] M. Zhao and G. S. Zhou, “Estimation of biomass and net primary productivity of major planted forests in China based on forest inventory data,” *For. Ecol. Manag.*, vol. 207, no. 3, pp. 295–313, 2005.

[7] E. Shang, E. Xu, H. Zhang, and F. Liu, “Analysis of spatiotemporal dynamics of the Chinese vegetation net primary productivity from the 1960s to the 2000s,” *Remote Sens.*, vol. 10, no. 6, 2018.

[8] G. A. Alexandrov, T. Oikawa, and Y. Yamagata, “The scheme for globalization of a process-based model explaining gradations in terrestrial NPP and its application,” *Ecol. Modell.*, vol. 148, no. 3, pp. 293–306, 2002.

[9] Q. Wang *et al.*, “The effects of air temperature and precipitation on the net primary productivity in China during the early 21st century,” *Front. Earth Sci.*, vol. 12, no. 4, pp. 818–833, 2018.

[10] Z. X. Guo, Z. M. Wang, D. W. Liu, K. S. Song, and C. C. Song, “Analysis of the influence factors of low-yield cropland in the Sanjiang Plain based on MOD17A3 dataset.,” *Trans. Chinese Soc. Agric. Eng.*, vol. volume 25, pp. 152–155, 2009.

[11] O. Gulbeyaz, B. Bondlamberty, Z. Akyurek, and T. O. West, “A new approach to evaluate the MODIS annual NPP product (MOD17A3) using forest field data from Turkey,” *Int. J. Remote Sens.*, vol. 39, no. 8, pp. 2560–2578, 2018.

[12] X. C. Wang, S. D. Wang, and H. B. Zhang, “Spatiotemporal pattern of vegetation net primary productivity in Henan Province of China based on MOD17A3,” *Chinese J. Ecol.*, vol. 32, no. 10, pp. 2797–2805, 2013.

[13] C. B. Field, J. T. Randerson, and C. M. Malmström, “Global net primary production: Combining ecology and remote sensing ☆,” *Remote Sens. Environ.*, vol. 51, no. 1, pp. 74–88, 1995.

[14] X. Jia, B. Xie, M. Shao, and C. Zhao, “Primary Productivity and Precipitation-Use Efficiency in Temperate Grassland in the Loess Plateau of China,” *PLoS One*, vol. 10, no. 8, p. e0135490, 2015.

[15] M. Khalifa, N. A. Elagib, L. Ribbe, and K. Schneider, “Spatio-temporal variations in climate, primary productivity and efficiency of water and carbon use of the land cover types in Sudan and Ethiopia,” *Sci. Total Environ.*, vol. 624, pp. 790–806, 2017.

[16] D. W. Kicklighter, A. Bondeau, A. L. K. J. Schloss, and A. D. Mcguire, “Comparing global models of terrestrial net primary productivity (NPP):global pattern and differentiation by major biomes,” *Glob Chang. Biol*, vol. 5, no. S1, pp. 16–24, 2010.

[17] B. Liu, X. Chen, Q. Meng, H. Yang, and J. Van Wart, “Agricultural and Forest Meteorology Estimating maize yield potential and yield gap with agro-climatic zones in China — Distinguish irrigated and rainfed conditions,” *Agric. For. Meteorol.*, vol. 239, pp. 108–117, 2017.

[18] Q. Zu, C. Mi, D. L. Liu, L. He, Z. Kuang, and Q. Fang, “Spatio-temporal distribution of sugarcane potential yields and yield gaps in,” vol. 92, no. October 2017, pp. 72–83, 2018.

[19] Z. Xinke, L. Luo, and X. Xinliang, “Temporal and spatial variation characteristics of climate production potential of maize in China in recent 30 years,” pp. 94–101, 2012.

[20] X. Zhang, “PE(possible evapotranspiration) index of vegetation and vegetation-climate classification (I) -- introduction to several main methods and PEP procedures,” *J. plant Ecol.*, vol. 13, no. 1, pp. 197–207, 1989.

[21] “A preliminary study on the net primary productivity model of natural vegetation,” *J. plant Ecol.*, vol. 19, no. 3, pp. 193–200, 1995.

[22] C. S. Potter *et al.*, “Terrestrial ecosystem production: A process model based on global satellite and surface data,” *Global Biogeochem. Cycles*, vol. 7, no. 4, pp. 811–841, 1993.

[23] H. Lieth, *Modeling the Primary Productivity of the World*. 1975.

[24] Z. H. Chen *et al.*, “Estimation of Heihe Basin Net Primary Productivity Using the CASA Model,” *J. Nat. Resour.*, 2008.

[25] K. Yin, Y. Tian, C. Yuan, F. Zhang, Q. Yuan, and L. Hua, “NPP spatial and temporal pattern of vegetation in Beijing and its factor explanation based on CASA model,” *Remote Sens. L. Resour.*, vol. 27, no. 1, pp. 133–139, 2015.

[26] K. Chen, Y. Han, S. Cao, J. Ma, G. Cao, and H. Lu, “The Study of Vegetation Carbon Storage in Qinghai Lake Valley Based on Remote Sensing and CASA Model,” *Procedia Environ. Sci.*, vol. 10, no. Part B, pp. 1568–1574, 2011.

[27] P. Shi-Long and Q. H. Guo, “APPLICATION OF CASA MODEL TO THE ESTIMATION OF CHINESE TERRESTRIAL NET PRIMARY PRODUCTIVITY,” *Acta Phytoecol. Sin.*, vol. 25, no. 5, 2001.

[28] W. Zhu, Y. Pan, and J. Zhang, “Estimation of net primary productivity of chinese terrestrial vegetation based on remote sensing (In Chinese),” *Chinese J. Plant Ecol.*, vol. 31, no. 3, pp. 413–424, 2007.

[29] Q. Sun, C. Miao, and Q. Duan, “Extreme climate events and agricultural climate indices in China: CMIP5 model evaluation and projections,” *Int. J. Climatol.*, vol. 36, no. 1, pp. 43–61, 2016.

[30] S. Chen *et al.*, “Evaluation of the successive V6 and V7 TRMM multisatellite precipitation analysis over the Continental United States,” *Water Resour. Res.*, vol. 49, no. 12, pp. 8174–8186, 2013.

[31] H. Guo *et al.*, “Early assessment of Integrated Multi-satellite Retrievals for Global Precipitation Measurement over China,” *Atmos. Res.*, vol. 176–177, pp. 121–133, 2016.

[32] P. Schubert *et al.*, “Remote Sensing of Environment Modeling GPP in the Nordic forest landscape with MODIS time series data — Comparison with the MODIS GPP product,” *Remote Sens. Environ.*, vol. 126, pp. 136–147, 2012.

[33] D. P. Turner *et al.*, “Evaluation of MODIS NPP and GPP products across multiple biomes,” vol. 102, pp. 282–292, 2006.

[34] C. Shim, J. Hong, J. Hong, Y. Kim, and M. Kang, “ScienceDirect Evaluation of MODIS GPP over a complex ecosystem in East Asia : A case study at Gwangneung flux tower in Korea,” *Adv. Sp. Res.*, vol. 54, no. 11, pp. 2296–2308, 2014.

[35] T. Luo, “Biological productivity patterns and mathematical models of major forest types in China,” 1996.

[36] J. He, P. Zhang, W. Jing, and Y. Yan, “Spatial Responses of Net Ecosystem Productivity of the Yellow River Basin under Diurnal Asymmetric Warming,” *Sustainability*, vol. 10, no. 10, p. 3646, 2018.

[37] J. Li *et al.*, "Response of net primary production to land use and land cover change in mainland China since the late 1980s," *Sci. Total Environ.*, vol. 639, pp. 237–247, 2018.

[38] C. ming SUN, X. chun ZHONG, C. CHEN, T. GU, and W. CHEN, "Evaluating the grassland net primary productivity of southern China from 2000 to 2011 using a new climate productivity model," *J. Integr. Agric.*, vol. 15, no. 7, pp. 1638–1644, 2016.

[39] WeiZHOU, ZhengGuoSUN, JianLongLI, ChengChengGANG, and ChaoBinZHANG, "Desertificationdynamicandtherelativerolesofclimatechangeandhumanactivitiesindesertification intheHeiheRiverBasinbasedonNPP," *J. Arid Land*, vol. 5, no. 4, pp. 465–479, 2013.

[40] F. Gu *et al.*, "Climate-driven uncertainties in modeling terrestrial ecosystem net primary productivity in China," *Agric. For. Meteorol.*, vol. 246, no. June, pp. 123–132, 2017.

[41] F. Fenga *et al.*, "Net primary productivity of China's terrestrial ecosystems from a process model driven by remote sensing," *J. Environ. Manage.*, vol. 85, pp. 563–573, 2007.

[42] F. Pei, X. Li, X. Liu, and C. Lao, "Assessing the impacts of droughts on net primary productivity in China," *J. Environ. Manage.*, vol. 114, pp. 362–371, 2013.

[43] W. Jiao, Y. Chen, W. Li, C. Zhu, and Z. Li, "Estimation of net primary productivity and its driving factors in the Ili River Valley, China," *J. Arid Land*, vol. 10, no. 5, pp. 781–793, 2018.

[44] W. Zhu, Y. Pan, and J. Zhang, "Remote sensing estimation of net primary productivity of terrestrial vegetation in China," *J. plant Ecol.*, vol. 31, no. 3, pp. 413–424, 2007.

[45] I. P. Holman, "A soil-based approach to rainfall-runoff modelling in ungauged catchments for England and Wales Supervisor," *Cranf. Univ.*, 2004.

[46] J. Zhao, X. Yan, J. Guo, and G. Jia, "Evaluating Spatial-Temporal Dynamics of Net Primary Productivity of Different Forest Types in Northeastern China Based on Improved FORCCHN," *PLoS One*, vol. 7, no. 11, pp. 1–14, 2012.

[47] D. P. Turner *et al.*, "Site-level evaluation of satellite-based global terrestrial gross primary production and net primary production monitoring," *Glob. Chang. Biol.*, vol. 11, no. 4, pp. 666–684, 2005.

Manuscript_Revise

Click here to access/download;Manuscript;1.Evaluation of NPP using three models compared over China.docx

Formatted: Border: Bottom: (No border)

Evaluation of NPP using three models compared with MODIS-NPP data over China

Jinke Sun[1], Dan Cao[2], Haipeng Niu[1]~~, Haitao Jing[1]~~

[1]School of Surveying and Land Information Engineering, Henan Polytechnic University, Jiaozuo, Henan, China

[2]Key Laboratory of Digital Earth Science, Institute of Remote Sensing and Digital Earth, Chinese Academy of Sciences, Beijing, China;

Correspondence author: Haipeng Niu (Email: niuhaipeng@126.com)

ABSTRACT ~~Estimating net primary productivity (NPP) is a crucial component in global climate change researches and carbon cycling.~~ Estimating net primary productivity (NPP) is crucial in global climate change research and carbon cycling. However, there are many uncertainties ~~in~~ different NPP modeling results and the process of~~of~~ NPP is ~~difficult~~ challenging to model ~~in~~ on the absence of data. In this study, we only used meteorological data as input, the estimated NPP using climate productivity model, synthetic model and CASA model were compared with MOD17A3-NPP as the reference data over China during the past 16 years (2000-2015). Then the statistics evaluation metrics (Relative Bias (RB), Pearson linear Correlation Coefficient (CC), Root Mean Square Error (RMSE), and Nash-Sutcliffe efficiency coefficient (NSE)) for three models with the reference data were calculated. The correlation analysis implied that the CASA-model performed better than the other two models in terms of RB, RMSE, NSE and CC whether on the national or the regional scale. It has a higher CC with 0.5081 and a smaller RMSE with 111.96 g C·m^{-2}·yr^{-1} in the whole country. The synthetic model and CASA-model has~~have~~ the same advantages at some regions, and the artificial~~synthetic~~ model has lower RMSE in Southern China (86.35 g C·m^{-2}·yr^{-1}), Xinjiang (85.53 g C·m^{-2}·yr^{-1}) and Qinghai-Tibet Plateau (93.22 g C·m^{-2}·yr^{-1}) than the others. The climate productivity model has widespread overestimation and large systematic error, along with worse performances (NSEmax=0.45) and other metric indexes unsatisfactory, especially Qinghai-Tibet Plateau have~~has~~ relatively lower accuracy because of the available observation data is insufficient. The results ~~in~~ from different regions also illustrated that different models for NPP estimating was suitable for ~~different~~various other ~~regions~~areas. Overall, the CASA-model is much more ~~suitable~~ ideal for estimating NPP all over China in the absence of data. This study provides a comprehensive intercomparison ~~intercomparsion~~ of remote sensing, based on national NPP estimation, and anticipates future NPP simulation development ~~development of NPP simulation~~ at the national and global scale.

Keywords : NPP estimation, climate productivity model, Synthetic model, CASA-model, MOD17A3, statistics evaluation

I. Introduction

~~Vegetation net primary productivity (NPP),~~ NPP is the amount of organic matter produced by photosynthesis minus autotrophic respiration, which is defined as the net amount of organic matter fixed by plants through photosynthesis. It represents the net carbon flow from the atmosphere to the terrestrial ecosystems and is affected by many factors, such as climate, soil, nutrients and CO_2 [1]–[3]. Generally, assuming that vegetation can make full use of the climate resources, such as light, heat ,and water when other factors are in the optimum state, which can obtain the maximum biological or agricultural yield per unit area of land is called climatic production potential[4], [5]. As one of the ~~important~~ critical indicators of ecosystem function, NPP reflects the growth of vegetation and the health status of ecosystems [6] as well as changes in climate and ecosystem function change [7], and it is useful in modeling researches of regional and global carbon and water cycles. Therefore, a better understanding of NPP estimates is ~~important~~ essential for predicting the future carbon flux of terrestrial ecosystems in the context of global warming.

Traditionally, NPP estimations were based on field surveys and fixed-point observations. However, these methods are not feasible on a large scale because of its low efficiency, high cost, and inability to obtain complete spatial coverage and continuous long-term time series [8], [9]. With the development of satellite remote sensing technology, we can find a powerful way to retrieve NPP's spatial distribution inland vegetation. ~~With the development of satellite remote sensing technology, we can find a powerful way to retrieve the spatial distribution~~

Formatted: Border: Bottom: (No border)

of NPP in land vegetation. MOD17A3-NPP, as one existing large scale product acquired by remote sensing, has 1 km resolution and wide complete applications in different ecosystems and is often recognized as better validation data for the large-scale spatial coverage [10] – [12]. Additionally, more and more scholars estimated vegetation NPP using remote sensing models in different regions of the world as well as analyzed its temporal and spatial distribution features from different angles [7], [13] – [16]. Generally, there are large uncertainties due to the lack of data in the large-scale NPP calculation. [17]–[19]. Therefore, models that require fewerless data have a greater more significant advantage over large-scale for NPP simulation, such as climate productivity models (e.g. Miami model (Lieth, 1975), Thornthwaite Memorial model (Lieth, 1975)), synthetic model [20], [21], light to use efficiency models (e.g. CASA modlesmodel [22]) and so on. Climate productivity models are always used to estimate potential productivity as the maximum regional productivity [1].In previous studies, many researchers also chose the climate productivity model [23] and synthetic model [20], [21] to estimate the potential productivity [1]. The synthetic artificial model is established mainly based on the measured biomass data, which is from 125 stations connected with natural mature and 23 stations related to natural vegetation NPP in China such as grassland, and desert [6]. Meanwhile, there is a notable advantage for estimating actual productivity based on the CASA model as a light use efficiency models [24]–[27] and the current version of the CASA model take into account land-cover change[28]. However, there are many differences and uncertainties during these models. Systematic validation of those models is rare in large-scale regions, which may influence our understanding of the ecosystem's carbon balance and assess vegetation response to climate. Systematic validation of those models is rare in large-scale regions, which may influence our understanding of the ecosystem carbon balance and assessment of vegetation response to climate. Moreover, most studies have focused on a single NPP estimated model and the results of various methods used to have considerable differences inwith uncertainty. Evaluating the reliability of results acquired by different models and assessing the differences are still open questions. How to evaluate the reliability of results acquired by different models and how to assess the differences are still open questions.

In this study, we only used meteorological data as input data, the estimated NPP using climate productivity model (Thornthwaite Memorial model), synthetic model and CASA model were compared with MOD17A3-NPP as the reference data over China during the past 16 years (2000-2015). Then the statistics evaluation metrics (Relative Bias (RB), Pearson linear Correlation Coefficient (CC), Root Mean Square Error (RMSE), and Nash-Sutcliffe efficiency coefficient (NSE)) for three models with the reference data were calculated. The study results will provide baseline data for large-scale and long-time series NPP evaluation and help the policymakers understand the current situation of NPP spatial distribution in China and develop environmental policies related to crop production.The study results will not only provide baseline data for large-scale and long-time series NPP evaluation, but also will help the policymakers to understand the current situation of NPP spatial distribution in China, and develop environmental policies related to crop production.

II. Materials and methods

A. Study area

China, the third-largest county globally, is located in the East of Asia on the western shore of the Pacific Ocean.

China, the third-largest county in the world, locates in the East of Asia on the western shore of the Pacific Ocean. China spans tropical, subtropical, warm temperate, middle temperate and cold temperate climate zones from south to north. Temperate Mild monsoon climate, subtropical monsoon climate, tropical monsoon climate, tropical rainforest climate, temperate continental climate and plateau mountain climate dominate different regions in China, and the diversity of climate types results in various vegetation types[29]. To clearly to distinguish NPP in China under different other climatic and topographic conditions, we divide China into seven major regions and each of which may contain one or more administrative regionsareas. The seven major significant regions include：Ⅰ) the Xinjiang (XJ) region, which has arid and semi-arid climate characteristics. Ⅱ) Qinghai – Tibet Plateau (TP) which has an average elevation of about 4500 m. Ⅲ) Northwestern China (NW) bounded by the 400 mm annual precipitation isohyet. Ⅳ) Northeastern China (NE) is located in the north of the Yan mountains. Ⅴ) Northern China (NC) is located in the north of the Qinling Mountains–Huai River line and the vegetation patterns in this region are characterized by a mosaic of agricultural vegetation. VI) Yunnan–Guizhou Plateau in southwestern China (SW) which is bounded by the Ta-pa Mountains and Wulingshan Mountains to the north and east. VII) and Southern China (SC) south of the Nanling Mountains and southeast Southeast of the Wuyi Mountains. Southern China (SC) south of the Nanling Mountains and southeast Southeast of the Wuyi Mountains. The same China divisionsthe same divisions of China are from [30], [31] .These subregions abbreviations are labeled in Fig1a and will be used herein.

Formatted: Border: Bottom: (No border)

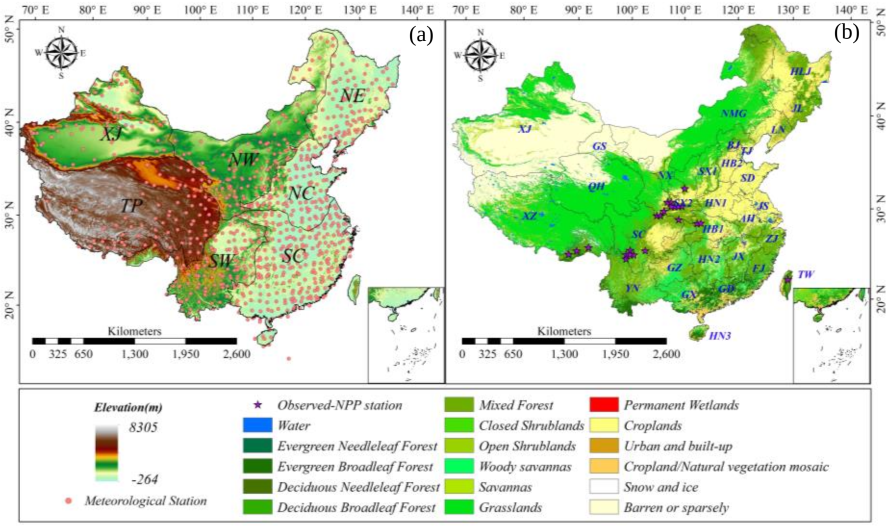

Fig. 1 (a) Different regions of the study area according to the elevation and annual precipitation. The pink circles are 839 meteorological stations. (b) Land use of China. The 23 stars represent the observed-NPP stations from published papers, and these stations located in the temperate forest are mainly used to verify the potential productivity

B. Data and processing

1) REMOTE SENSING DATA

a. MODIS NPP data

The MOD17A3's global NPP data for 2000-2015 ~~were~~ was used as the reference data in this study. These data were obtained from NASA's website (https://lpdaac.usgs.gov/data_access/) with 1 km spatial resolution. It contains total primary productivity (GPP), net primary productivity (NPP) and net ~~primary~~ direct quality control (NP_QC). In this study, 21 images in China were selected. The NPP data from 2000 to 2015 were converted into NPP data with unit g C·m^{-2}·yr^{-1}, and the scale coefficient was 0.1(Fig.2). Studies found that MOD17A3 NPP dataset has ~~very good~~outstanding agreements with the observations at the global or country scale[32]–[34].

b. MODIS NDVI data

MODIS normalized difference vegetation indexes~~index~~ (NDVI) product with a 250m/16-day spatiotemporal resolution between 2000 and 2015 was used in this study. These data were from the MODIS product MOD13A1. ~~The monthly NDVI data were generated by the maximum-value composite method and then were reprojected to the Albers equal-area projection.~~ The maximum-value composite method generated the monthly NDVI data and then were reprojected to the Albers equal-area projection. The NDVI data were then used to drive CASA-model as input data for NPP estimation.

2) METEOROLOGICAL DATA

This study used meteorological data such as daily temperature, daily precipitation, and solar radiation from 2000 to 2015. All data is provided by the China Meteorological Administration (http://cdc.nmic.cn/home.do), obtained from the 839 meteorological stations (130 solar radiation stations) in the whole country. Meteorological data driving the CASA model include monthly precipitation, monthly mean temperature, and monthly solar radiation. The data supplied to drive the Thornthwaite Memorial and Zhou model are only yearly temperatures and precipitation datasets. These data were interpolated using ANUSPLIN (version 4.2) to create regular monthly data or yearly data layers with the same spatial resolution as MOD17A3-NPP.~~These data were interpolated using ANUSPLIN (version 4.2) to create regular monthly data or yearly data layers with the spatial resolution the same as MOD17A3-NPP.~~

Formatted: Border: Bottom: (No border)

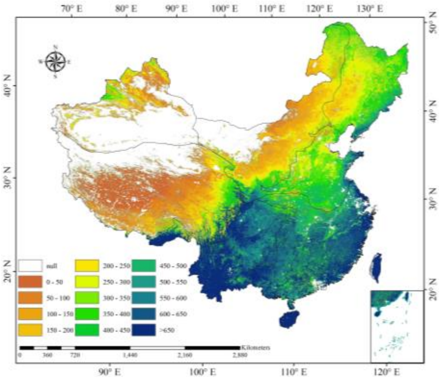

Fig. 2 The spatial distribution of multi-year MOD17A3-NPP average from 2000 to 2015

3) LAND USE DATA AND OTHERS

Land use maps were from the MODIS product of MCD12Q1 and obtained by NASA (https://lpdaac.usgs.gov/data_access/). The data are classified into 14 categories according to IGBP global vegetation classification scheme, including the Evergreen Needleleaf Forest, Evergreen Broadleaf Forest, Deciduous Needleleaf Forest, Deciduous Broadleaf Forest, Mixed Forest, Closed Shrublands, Open Shrublands Woody Savannas, Savannas Grasslands, Permanent Wetlands, Croplands, Urban and built-up, Cropland/natural vegetation mosaic, Snow and Ice, and Barren or Sparsely land (Fig.1b). These data were also reprojected to the Albers equal-area projection. The datasets have been widely used in detecting land use/ land cover change worldwide and were used as input data in CASA-model.

DEM data with 1km resolution was from the Resource and Environment Data Cloud Platform, which was mainly used for This study used meteorological data such as meteorological data interpolation as affecting factors of temperature and precipitation.

4) IN-SITU SURVEY DATA

In this study, the verification NPP data were derived from the study of [35], with the Global Primary Productivity Initiative (https://daac.ornl.gov/). These data come from the National Forest Resources Inventory conducted by the Chinese Forestry Department during the period 1989-1993. Besides, we also used in-situ survey datasets from published kinds of literature with well-documented field sites [1], [36]–[38]. These data provided some valuable information such as site names, latitude, longitude, elevation, biomass and NPP estimations for most of the plant components. Especially Mostly, the data from Global Primary Productivity Initiative covered representative sample points of different vegetation types, and the data are widely used in global model parameterization and result verification. All data included different vegetation types and total NPP data of China's administrative districts. All data included not only different vegetation type, but also total NPP data of China's administrative districts. Finally, 23 observed-NPP stations (Fig.1b) and total unlimited amounts of every administrative district community were collected as NPP validation data nationwide.

C. Methods

1) CLIMATE PRODUCTIVITY MODEL FOR ESTIMATING NPP

Thornthwaite Memorial[39], as one climate productivity productivitu model, established a statistical relationship between Net Primary Productivity (NPP) and evapotranspiration (ET) based on the relationship between evapotranspiration, temperature, precipitation, and vegetation. On this basis, Lieth (1975) proposed the Thornthwaite Memorial model in 1974 based on the vegetation NPP in 50 different locations on 5 continents. The climate factors considered in this model are relatively simple and can better reflect the key factors affecting plant growth and development, such as temperature, precipitation, and evapotranspiration. The calculation formula is

$$NPP_T = 3000 \times [-e^{-0.0009695(v-20)}] \quad (1)$$

$$v = \frac{1.05r}{\sqrt{1 + (1 + \frac{1.05r}{L})^2}} \quad (2)$$

Formatted: Border: Bottom: (No border)

$$L = 300 + 25T + 0.05T^3 \quad (3)$$

where, v: Annual average actual evapotranspiration (mm), r: Annual average precipitation (mm), L: the maximum annual evapotranspiration (mm), expressed as a function of the average annual temperature T (℃). NPP_T is the climate productive potential of vegetation determined by evapotranspiration that was calculated in units of g DW/m^2/yr. This was implemented by applying a conversion factor of 0.475 [28] in China from dry matter (DW) to carbon content (g C·m^{-2}·yr^{-1}).

2) SYNTHETIC MODEL FOR ESTIMATING NPP

According to the physiological and ecological characteristics of plants and the regional evapotranspiration model based on the energy balance equation and the water balance equation, Zhou (1995) and Zhang(1989) established the NPP model of natural vegetation combining the physiological and ecological characteristics of plants and the relationship between water and heat balance. The calculation formula [1] is as follows:

$$NPP = 100 \times RDI \frac{rR_n(r^2 + R_n{}^2 + rR_n)}{(R_n + r)(R_n{}^2 + r^2)} exp(-\sqrt{9.87 + 6.25RDI}) \quad (4)$$

$$R_n = RDI \times r \times L \quad (5)$$

$$RDI = (0.629 + 0.237PER - 0.00313PER^2)^2 \quad (6)$$

$$PER = \frac{PET}{r} = \frac{BT \times 58.93}{r} \quad (7)$$

$$BT = \frac{\sum t_d}{365} \quad (8)$$

$$L = 597 - 0.6T_m \quad (9)$$

NPP was calculated in units of g DW·m^{-2}·yr^{-1} and was implemented by applying a conversion factor of 0.475 in China from dry matter (DW) to carbon content (g C·m^{-2}·yr^{-1}). r is average annual precipitation, R_n is annual net radiation, RDI is radiant dryness, L is the latent heat of evaporation, PET is potential evapotranspiration, PER is probably evapotranspiration rate. Biological temperature (BT) is the average temperature experienced during plant growth, generally between 0 and 30 ℃. Mean daily temperature (t_d) and mean monthly temperature (T_m) takestake 0 ℃ when it lowerslower than 0 ℃ and can be calculated at 30 ℃ when the temperature is higher than 30 ℃.

3) CASA-MODEL FOR ESTIMATING NPP

The Carnegie-Ames-Stanford Approach (CASA) models[13], [22], a satellite-based photosynthetic utilization models, is widely used to calculate the NPP. The CASA model requires the following parameters, such as temperature, rainfall, solar radiation, NDVI, etc. CASA model requires the following parameters, such as temperature, rainfall, solar radiation, NDVI and so on. The model can be calculated by APAR (Absorbed Photosynthetic Active Radiation) times the light energy conversion rate ε. The calculation expression is as follows:

$$\mathrm{NPP}(x,t) = \mathrm{APAR}(x,t) \times \varepsilon(x,t) \quad (10)$$

$$\mathrm{APAR}(x,t) = \mathrm{FAPAR}(x,t) \times \mathrm{PAR}(x,t) = \mathrm{SOL}(x,t) \times \mathrm{FAPAR}(x,t) \times 0.5 \quad (11)$$

where, NPP(x,t) represents NPP fixed by vegetation at a grid cell x in month t. APAR(x,t) represents photosynthetically active radiation absorbed by green vegetation. ε(x,t) represents actual the utilization of light energy. SOL(x,t) represents the total solar radiation of pixel x in month t. PAR is the incident photosynthetically active radiation (M J m-2) per month. FPAR(x,t) is the fraction of PAR absorbed by the vegetation canopy. Constant 0.5 represents the solar effective radiation ratio that vegetation can utilize, namely, the ratio of PAR divided by SOL. Constant 0.5 represents the solar effective radiation ratio that can be utilized by vegetation, namely, the ratio of PAR divided by SOL. The detail of the CASA-model can be found in the study of Potter (1993) and Zhu (2007).

4) VALIDATION AND STATISTICAL EVALUATION METRICS

In this study, we validated the estimated NPP by comparing it with measured data. These data were collected from Luo's investigation data[35] and other published literature. We are also the focus on the evaluation of different results from three models (Thornthwaite Memorial model, Synthetic model and CASA-model) in this study. The average result of MOD17A3-NPP was used as the reference simulation compared with the NPP result using three models. In addition, we are the focus on the evaluation of different results from three models (Thornthwaite Memorial model, Synthetic model and CASA-model) in this study. The average result of MOD17A3-NPP was used as the reference simulation compared with the result of NPP estimated using three models. A series of

Formatted: Border: Bottom: (No border)

traditional error indexes, which include Bias, Relative Bias(RB), Pearson linear Correlation Coefficient (CC), Root Mean Square Error (RMSE), and Nash-Sutcliffe efficiency coefficient (NSE), is calculated at a pixel in this study. RB, NSE ,and CC are dimensionless, and RMSE is in gC/m^2. RB, when multiplied by 100, denotes the degree of overestimation or underestimation in percentage. The definition of RB, CC, and RMSE can be found in [30], [31], [40], and NSE that is generally used to verify the quality of the hydrological model simulation results are defined as follows:

$$NSE = 1 - \frac{\sum_{t=1}^{T}\left(Q_0^t - Q_m^t\right)^2}{\sum_{t=1}^{T}\left(Q_0^t - \bar{Q}_0\right)^2} \quad (12)$$

Q_0 : the result of the reference model; Q_m : the simulated value of the comparison model;

Q_0s: the annual mean of the model data;

t :the year;

The value range of *NSE* is (-∞, 1), and the closer the value is to 1, the higher the similarity between the comparison models and the reference models areis. The closer the value is to 0, the closer the simulation result of the comparison model is to the result of the reference model, that is, the more reliable the overall result is., but the process simulation error of that is large. When the value of *NSE* is far less than 0, it indicates that the model is not credible [40].

III. Results and discussion

A. Validation of estimated NPP

In this study, we validated the estimated NPP at the provincial scale (Fig.3a and 3b) and the station scale (Fig.3c and 3d), respectively. The results showed that the simulated average annual NPP in each province from 2000 to 2015 using the three models (Thornthwaite Memorial model, Synthetic model and CASA-model) in this study are is highly correlated with the result of Luo during 1989 to 1993 (Fig.3b). R^2 was 0.5188, 0.6653 and 0.692, respectively, and the corresponding RMSE was 1.67 $t\cdot ha^{-1}\cdot yr^{-1}$, 0.6653 $t\cdot ha^{-1}\cdot yr^{-1}$ and 0.692 $t\cdot ha^{-1}\cdot yr^{-1}$, respectively. However, the Thornthwaite Memorial model and Synthetic model's annual average values are higher whereas the CASA-model are lower than those of Luo (Fig.3a) at the provincial scale. However, the annual average values of the Thornthwaite Memorial model and Synthetic model is higher whereas the CASA-model are lower than those of Luo (Fig.3a) at the provincial scale. It may be caused by various uncertainties such as time inconsistency and the former two models are used to estimated potential productivity. In addition, we also compared MODIS NPP products with the observed data. The results (R^2=0.81) showed that MODIS NPP products can meet the needs of long time series NPP variation. . Fig. 3c and 3dalso illustrated CASA model was better than the other two models at the station scale and the results of CASA model were more closer MODIS NPP products, which is likely due to CASA-model and MOD17A3 products both belong to the light use efficiency(LUE) model. The validation results provide a basis for comparing the subsequent results on grid-scale with reference data. Besides, the total NPP of China land simulated by the Thornthwaite Memorial model, Synthetic model and CASA-model were 4.03Pg C (1Pg C=10^{15}g C), 2.47 Pg C and 3.58 Pg C, respectively. This is within the reported values of 1.95-6.13 Pg C[28], [41], [42]. We also compared the NPP calculated from the three models with other simulation results (Table 1). This indicated the reliability of our results.

Table 1 Comparison of annual average NPP between this study and other studies

Reference	Studied period	Studied area	model	Precision
This study	2000-2015	China	TW model	R^2 = 0.61
			ZGS model	R^2 = 0.76
			CASA model	R^2 = 0.78
(Zhou et al., 2013)	2001-2010	Heihe River Basin	TW model	None
[1]	2000-2015	The Loess Plateau	ZGS model	R^2 = 0.734, P <0.01
[1]	2000-2015	The Loess Plateau	CASA model	R^2 = 0.817, P <0.01
[42]	2001-2010	China	CASA model	r=0.733,P<0.001
[43]	2000-2014	The Ili River Valley	CASA model	R^2 = 0.65, P <0.01,
(Zhu et al., 2007)	1982-2000	China	CASA model	RB=4.5%

Formatted: Centered

Note: For charting and table simplification, TW represents the climate productivity (Thornthwaite Memorial) model. ZGS represents Synthetic model proposed by Guangsheng Zhou, the same below. The R2 at station scale used in this table.

Formatted: Border: Bottom: (No border)

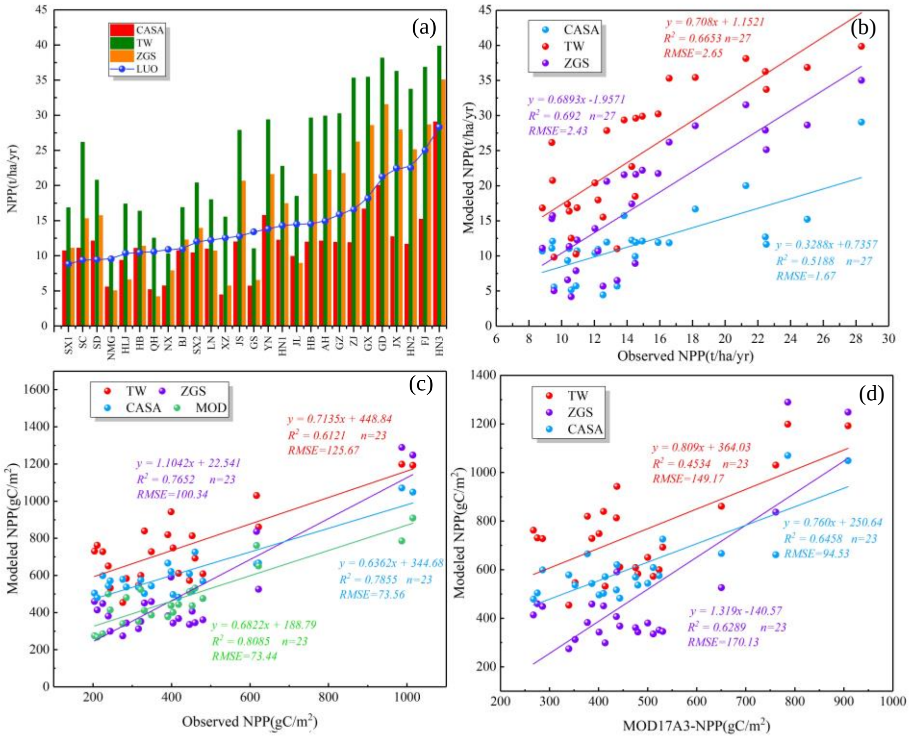

Fig.3 Validation ~~Validtion~~ of NPP (a). The range of NPP for three models and LUO's measured data in 27 provincial administrative regions (b). Validation of NPP for three models and LUO's measured data in 27 provincial administrative regions(R2 and RMSE) (c). Validation of NPP for three models and reference data MOD17A3 in 23 observed stations (R2 and RMSE) (d). Validation of NPP for three models and reference data MOD17A3 in 23 observed stations (R2 and RMSE).

B. Spatial distribution of NPP

Fig.4 showed that the spatial distribution of estimated NPP was~~were~~ consistent with those of the reference data. The general trend of NPP distribution in China is higher in the Southeast and lower are~~in~~ the northwest. The spatial distribution of productivity varies from year ~~to~~ after year due to different climatic factors, topographic factors, phenological characteristics , and vegetation types. As shown in Fig.4, there are ~~obvious~~ pronounced regional differences in every model, ~~showing~~ offering a gradually decreasing trend from ~~southeast~~ Southeast to northwest. In southern China, the evergreen broad-leaved forest is widely distributed and rich in resources. The annual average NPP is higher than 600 g C·m^{-2}·yr^{-1}. Rich precipitation groundwater and a warm climate are more conducive to the growth of vegetation [9]. However, the average annual NPP is lower than 200 g C·m-2·yr-1 in the northwest due to poor soil, low temperatures and low rainfall [28]. Overall, the maximum value of NPP during 2000-2005, occurred in the southwest, southern China and Taiwan. The values between 600 and 700 g C·m^{-2}·yr^{-1} were located in the south of the lower reaches of the Yangtze River, east of Yunnan-Guizhou Plateau and north of Nanling Mountains with annual NPP. Annual NPP between 400 and 600 g C·m^{-2}·yr^{-1} were located in Daxing'an Mountains, Xiaoxing'an Mountains, east of Taihang Mountains, middle reaches of the Yangtze River Basin, most area of Sichuan, southeastern Tibet, Tianshan Mountains in Xinjiang and Altai Mountains with. The low-value ~~areas~~ sites mainly distribute in Inner Mongolia, Xinjiang, Qinghai-Tibet Plateau, and parts of Shanxi, Gansu, Ningxia and Shanxi provinces with annual NPP less than 200 g C·m^{-2}·yr^{-1}.

Formatted: Border: Bottom: (No border)

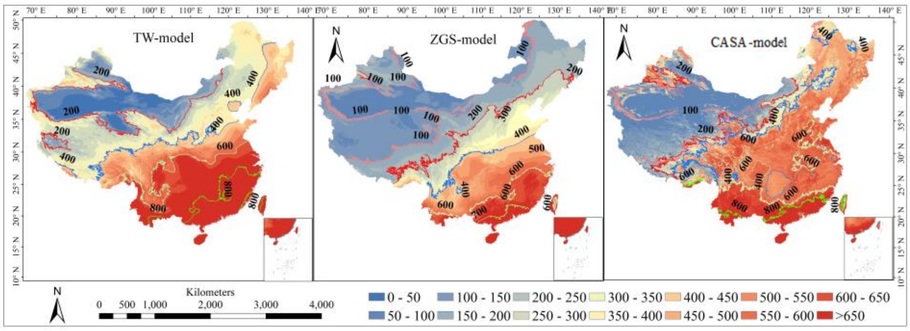

Fig.4 The spatial distribution of NPP from three models

However, there was also a discrepancy in different models. Spatial patterns of 16 years average NPP over China depicted by CASA-model, on the other hand, agree with the reference MOD17A3. The difference is that the Thornthwaite Memorial model and Synthetic models simulation results showed obvious banded distribution from northwest to ~~southeast~~Southeast, and the NPP contour increased steadily from northwest to ~~southeast~~Southeast, which was relatively smooth. The simulation results of the CASA model showed a larger zigzag shape, especially in the southern region, the simulation results had no obvious regional regularity. Additionally, the Thornthwaite Memorial model, a climate productivity potential model~~, which was obviously overestimated compared with the other two models and noted that the results of Thornthwaite Memorial model~~as obviously overestimated compared with the other two models and noted that the Thornthwaite Memorial model results reveal very high maxima at individual pixels especially in southern China. The ~~result of the Synthetic model~~Synthetic model's result showed close spatial patterns with the Thornthwaite Memorial model than those of the CASA-model in the contour line, and the contour lines of the Synthetic model showed obvious hysteresis phenomenon in the northwest-southeast direction. Synthetic model and CASA-model consider the influence of evapotranspiration on the model simulation results in the parameter input process~~, but the original input data of Synthetic model is the same as the Thornthwaite Memorial model, while~~. Still, the original input data of onto Synthetic model areis the same as the Thornthwaite Memorial model. At the same time, CASA-model takes into account the effects of radiation and land to use category on the results. Therefore, the results of CASA-model are closer to actual productivity.

Table 2 showed the relative bias (RB) between the annual average NPP of the three models and the reference data. Interestingly, the overall relative error is the Synthetic model (18.54%) less than CASA-model (21.34%) and the Thornthwaite Memorial model (30.34%). It indicated that the Synthetic model was underestimated whereas CASA-model and Thornthwaite Memorial model overestimated from the country scale. The relative precision decreased from 81.47% in the Synthetic model to 78.66% in CASA-model and 69.66% in the Thornthwaite Memorial model. Regionally, the ~~results of the Thornthwaite Memorial model~~Thornthwaite Memorial model results were overestimated in most regions, and the relative deviation are~~is~~ very high expectations for the region of SW (8.08%), which was only higher than that of CASA-model (6.86%) simulation in this region. The results of CASA-model were also largely overestimated compared with the reference data in most regions, especially in XJ, TP , NW, arid areas of Northwest China and plateau areas. The annual average estimation NPP of Synthetic models on~~in~~ SC, NC, TP, and NW were in good agreement with the reference data, and the relative deviation in other regions ~~was~~ were more than 30%. . All in all, the Synthetic model and CASA-model had the same advantage in overall simulation accuracy, which can be used in the absence of data.

Table 2

RB for the results of three models comparable with reference data (Note: TW represents Thornthwaite Memorial model)

	MOD_NPP (g C/m^2)	TW_NPP (g C/m^2)	TW_RB (%)	ZGS_NPP (g C/m^2)	ZGS_RB (%)	CASA-NPP (g C/m^2)	CASA-RB (%)
SC	617.34	771.00	24.89	596.07	-3.45	682.98	10.63
NE	318.24	381.16	19.77	177.31	-44.28	357.95	12.47
NC	351.80	470.72	33.80	353.65	0.53	461.89	31.29
XJ	58.01	136.84	135.89	76.52	31.91	119.71	106.36
TP	109.24	334.50	206.21	114.17	4.51	223.48	104.58

Formatted: Border: Bottom: (No border)

SW	578.08	531.38	-8.08	338.64	-41.42	538.45	-6.86
NW	167.79	242.66	44.62	136.12	-18.87	285.67	70.25
ALL	314.36	409.75	30.34	256.07	18.54	395.73	21.34

C. Subregional statistics evaluation of NPP

NSE (Fig 5) for the simulation of three models showed the results from CASA-model was~~ere~~ more consistent with the reference data. This was likely due to the models of two results were belonged to the LUE model. However, the NSE of CASA-model was close to 0 or negative infinite values in a few areas, such as the Qinghai-Tibet Plateau, which indicated that there was inadaptable in CASA-model in this area. This is likely because the meteorological stations are few and unevenly ~~distribution~~distributed, resulting in the error of interpolation results. The maximum value of NSE from the Synthetic model can reach 0.73 in the northeast and south of NE and NW. And it is greater than 0.5 in most areas of SW, the eastern part of TP and the edge of Xinjiang. Some researches indicated that the performance levels were defined as follows: NSE > 0.65 = excellent, 0.65 - 0.5 = very good, 0.5 - 0.2 = good, and < 0.2 = poor [40], [45]. NSE in most areas of central NC and some areas of southern NC was near 0.5, which indicated that Synthetic model had certain applicability in these areas. However, the simulation results of some areas such as the central and western parts of TP, the edge parts of Xinjiang and the parts of NC and SC border appear near 0. Compared with the simulation results of the two models mentioned above, the NSE simulation results of the Thornthwaite Memorial model were less than 0.5, it illustrated that there were great differences between the simulation result of the Thornthwaite Memorial model and MOD17A3.

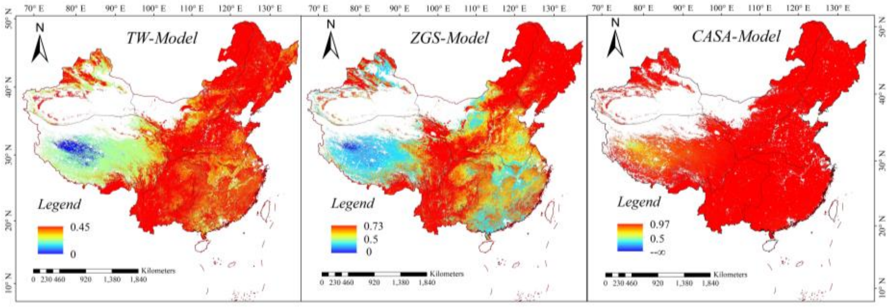

Fig.5 The spatial distribution of NSE for three models compared with reference dataset

The average RMSE (Fig 6) of the three models was less than 200 g C·m^{-2}·yr^{-1}, within the allowable range of their respective errors in the whole country. Table 3 indicated that CASA-model performed well noticeably in NE, XJ, SW and NW. The average of RMSE increased from 111.96 g C·m^{-2}·yr^{-1} in CASA-model to 133.14 g C·m^{-2}·yr^{-1} in Synthetic model and 172.46 g C·m^{-2}·yr^{-1} in Thornthwaite Memorial model. It showed the result from CASA-model ~~were~~ was consistent with the reference data. In terms of spatial distribution, the RMSE of CASA-model was ~~basically~~ 0-150 g C·m^{-2}·yr^{-1} in the whole region, especially in Inner Mongolia Autonomous Region, XJ, most areas of NE and Shandong Peninsula where RMSE was less than 50 g C·m^{-2}·yr^{-1}. RMSE was more than 200 g C·m^{-2}·yr^{-1} in the central of NC and TP. The larger RMSE occurs in southern SC, Southern SW and southern Tibet. However, the Synthetic model also had preponderance in most areas of NC, XJ and TP, and the range of RMSE was between 150-250 g C·m^{-2}·yr^{-1} in the northeastern margin area. The differences between simulated NPP and reference data were ~~larger~~ more considerable in most areas of SC and the junction area between TP and SC where RMSE was ~~greater~~ more significant than 400 g C·m^{-2}·yr^{-1}. Compared with the above two results, the RMSE of Thornthwaite Memorial model was higher in~~on~~ the whole country. In summary, the error of CASA-model and MOD17A3 reference data is the smallest, followed by Synthetic model, and the worst is Thornthwaite Memorial model.

Formatted: Border: Bottom: (No border)

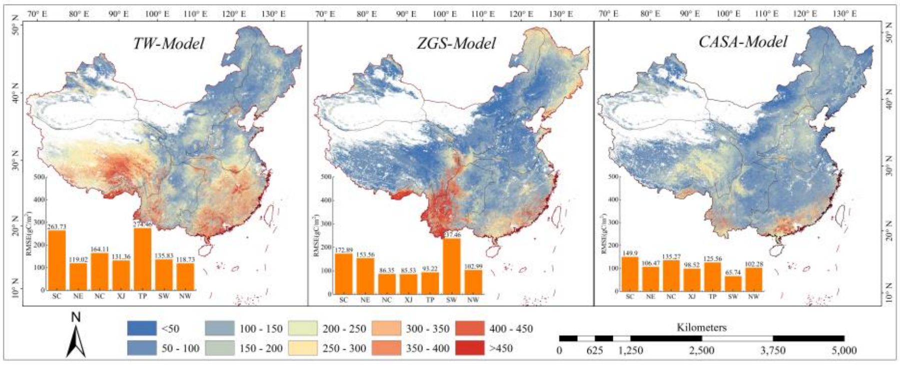

Fig.6 The spatial distribution of RMSE for three models compared with reference dataset

Table 3

RMSE of three models comparable with reference data

	TW-RMSE (g C/m^2)	ZGS-RMSE (g C/m^2)	CASA-RMSE (g C/m^2)
SC	263.73	172.89	149.9
NE	119.02	153.56	106.47
NC	164.11	86.35	135.27
XJ	131.36	85.53	98.52
TP	274.46	93.22	125.56
SW	135.83	237.46	65.74
NW	118.73	102.99	102.28
Average	172.46	133.14	111.96

CC in ~~CASA-model, Thornthwaite Memorial model and Synthetic model were not good consistent with those result in the reference data, and the average CC in~~the CASA-model, Thornthwaite Memorial model and Synthetic model were not good consistent with those resulting in the reference data. The average CC in the CASA-model was 0.5081 higher than CC in Synthetic model and ~~in~~ Thornthwaite Memorial model (Fig.7) in the whole country. However, the average values showed large regional differences. The CC of three models in ~~the region of NW and NE were~~NW and NE regions was ~~obviously~~ higher than the other regions. It illustrated that estimated NPP in these regions using CASA-model, Synthetic model and Thornthwaite Memorial model were well consistent with MOD17A3-NPP. However, the SC region captured the worst result in three models, as well as SW、TP、XJ and NC regions in the Thornthwaite Memorial model and Synthetic model except for CASA-model, which indicated that the models had a discrepancy in these areas. Fig.8 showed the differences of spatial distribution in three models were in different regions. In most areas of NE, NW, central NC and SW, the simulation results were better with those of reference data where CC can reach more than 0.8, showing a positive correlation. However, there was a negative correlation with CC between -1 and -0.8 in the southern part of TP and the border area between TP and SW, the eastern part of NE, the XJ boundary, Shandong Peninsula and the southern coastal area of SC. The overall performance of CASA-model is a positive correlation with MOD17A3 data. However, there was a negative correlation ~~which was lower than the above two models in most forest areas of NE~~that was lower than the above two models in most NE areas, most areas of TP and SC.

Formatted: Border: Bottom: (No border)

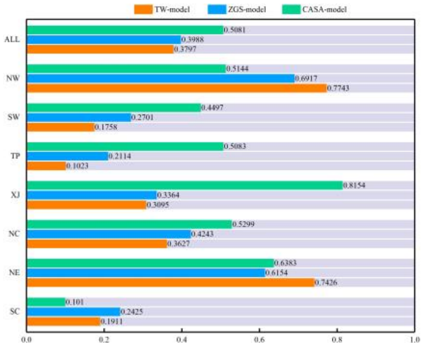

Fig.7 CC for three models compared with reference dataset

D. Uncertainties

The formation process of vegetation NPP is affected by many factors~~, which are~~ related not only to various physiological and ecological factors of the vegetation itself but also to many complicated environments. However, the three models estimation process and the MOD17A3-NPP used in this study take fewer factors into account~~, and t~~. The three models were all relatively simple, especially the MOD17A3-NPP was known to underestimate NPP in areas with high productivity, and overestimate NPP in low productivity areas [33]. There are still large uncertainties~~,~~ because the real situation was not entirely the same with the simulation. Due to the different physiological mechanisms of various ecosystems, such as forest ecosystem, grassland ecosystem and farmland ecosystem. Many scholars have proved there are obvious spatial-temporal variations between different vegetation types [7], [28], [41], [46], [47]. Although the validation data used in this study adopt the multi-year average of eliminating systemic errors, the inconsistency of time intervals will inevitably lead to errors. Additionally, the limitation of spatial ~~the resolution of remote sensing image data, a large number of mixed pixels are generated in the image, which affects the simulation results of NPP~~resolution of remote sensing image data, a large number of mixed pixels are generated in the image, which affects NPP's simulation results. Moreover, as for the multi-year NPP average, the uncertainty in simulated NPP also resulted from climate input data such as the differences in temperature, precipitation, topography and other aspects at the station scale through interpolated tools. Nonetheless, the accuracy of extraction results and the difference ~~from the three models are acceptable in the absence of data, and t~~between the three models are acceptable in the absence of data. This study provides new insight for large-scale and long-time series NPP evaluation and helps to understand the difference of various models.

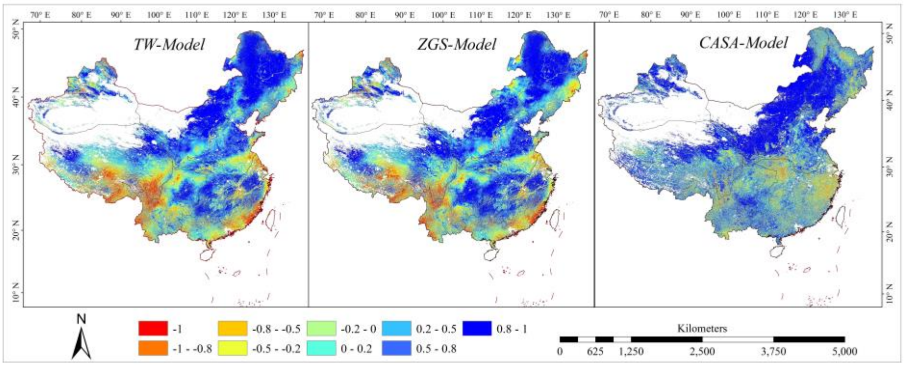

Fig.8 The spatial distribution of CC for three models compared with reference dataset

IV. Conclusions

In this study, NPP simulations of the climate productivity model, Synthetic model and CASA-model were compared within-site ~~with in-site~~ measured and MOD17A3-NPP as the reference data, and the multi-year NPP average from the three models were estimated in China during2000-2015. In addition, the statistics evaluation metrics (RB, RMSE, NSE, CC) for three models with the reference data were calculated. The results showed that

Formatted: Border: Bottom: (No border)

NPP simulations of the above three models were all within the reported values comparable to other's results from 2000 to 2015. The comparable results happened in the three models and reference data also illustrated that there are great differences. On the one hand, spatial patterns of NPP ~~decipted~~ decided by the Thornthwaite Memorial model, Synthetic model and CASA-model disagree with the reference MOD17A3-NPP. The results overestimated using the Thornthwaite Memorial model as a climate productivity model, ~~which is~~ mainly used to estimate potential productivity. The Synthetic model and CASA-model had same advantages from a regional perspective, and Synthetic model had lower RMSE in the region of SC (86.35 g $C\cdot m^{-2}\cdot yr^{-1}$), XJ (85.53 g $C\cdot m^{-2}\cdot yr^{-1}$) and TP (93.22 g $C\cdot m^{-2}\cdot yr^{-1}$) than others. On the other hand, CASA-model had ~~the~~ a great model goodness degree, correlation analysis and performed better in estimating actual NPP amounts than the other two models according to statistics evaluation metrics such as RB, RMSE, NSE and CC in the whole country. In addition, the difference between the three models occurred mainly in different ecosystems. ~~Note that t~~The three models revealed very high maxima at the individual pixels, especially in southeast China where there are a mixed forest, urban and built-up. In summary, the CASA-model agrees well with the reference data which can be used to estimated NPP in the absence of data. Importantly, these results can provide powerful help for researchers to select the appropriate NPP evaluation model.

V. Acknowledgments

The authors gratefully acknowledge the financial supports by the Innovative research team of Henan Polytechnic University (Grant No. T2018-4).

REFERENCES

[1] C. Gang, W. Zhao, T. Zhao, Y. Zhang, X. Gao, and Z. Wen, "The impacts of land conversion and management measures on the grassland net primary productivity over the Loess Plateau, Northern China," *Sci. Total Environ.*, vol. 645, pp. 827–836, 2018.

[2] N. J., "Estimating net primary productivity of grassland from field biomass measurements in temperate northern China," *Plant Ecol.*, vol. 174, pp. 217–234, 2004.

[3] H. Tian *et al.*, "Model estimates of net primary productivity, evapotranspiration, and water use efficiency in the terrestrial ecosystems of the southern United States during 1895–2007," *For. Ecol. Manag.*, vol. 259, no. 7, pp. 1311–1327, 2010.

[4] J. M. Liu and SHEN, "A dynamic division scheme of agro-climatic production potential for winter wheat in Beijing,Tianjin and Hebei province," *J. Meteorol. Environ.*, 2010.

[5] Z. Tian *et al.*, "Analysis of plant climatic production potential and it's restricting factors of 1 km2 grid of Beijing mountain area," *J. China Agric. Univ.*, 2004.

[6] M. Zhao and G. S. Zhou, "Estimation of biomass and net primary productivity of major planted forests in China based on forest inventory data," *For. Ecol. Manag.*, vol. 207, no. 3, pp. 295–313, 2005.

[7] E. Shang, E. Xu, H. Zhang, and F. Liu, "Analysis of spatiotemporal dynamics of the Chinese vegetation net primary productivity from the 1960s to the 2000s," *Remote Sens.*, vol. 10, no. 6, 2018.

[8] G. A. Alexandrov, T. Oikawa, and Y. Yamagata, "The scheme for globalization of a process-based model explaining gradations in terrestrial NPP and its application," *Ecol. Modell.*, vol. 148, no. 3, pp. 293–306, 2002.

[9] Q. Wang *et al.*, "The effects of air temperature and precipitation on the net primary productivity in China during the early 21st century," *Front. Earth Sci.*, vol. 12, no. 4, pp. 818–833, 2018.

[10] Z. X. Guo, Z. M. Wang, D. W. Liu, K. S. Song, and C. C. Song, "Analysis of the influence factors of low-yield cropland in the Sanjiang Plain based on MOD17A3 dataset.," *Trans. Chinese Soc. Agric. Eng.*, vol. volume 25, pp. 152–155, 2009.

[11] O. Gulbeyaz, B. Bondlamberty, Z. Akyurek, and T. O. West, "A new approach to evaluate the MODIS annual NPP product (MOD17A3) using forest field data from Turkey," *Int. J. Remote Sens.*, vol. 39, no. 8, pp. 2560–2578, 2018.

[12] X. C. Wang, S. D. Wang, and H. B. Zhang, "Spatiotemporal pattern of vegetation net primary productivity in Henan Province of China based on MOD17A3," *Chinese J. Ecol.*, vol. 32, no. 10, pp. 2797–2805, 2013.

[13] C. B. Field, J. T. Randerson, and C. M. Malmström, "Global net primary production: Combining ecology and remote sensing ☆," *Remote Sens. Environ.*, vol. 51, no. 1, pp. 74–88, 1995.

Formatted: Border: Bottom: (No border)

[14] X. Jia, B. Xie, M. Shao, and C. Zhao, "Primary Productivity and Precipitation-Use Efficiency in Temperate Grassland in the Loess Plateau of China," *PLoS One*, vol. 10, no. 8, p. e0135490, 2015.

[15] M. Khalifa, N. A. Elagib, L. Ribbe, and K. Schneider, "Spatio-temporal variations in climate, primary productivity and efficiency of water and carbon use of the land cover types in Sudan and Ethiopia," *Sci. Total Environ.*, vol. 624, pp. 790–806, 2017.

[16] D. W. Kicklighter, A. Bondeau, A. L. K. J. Schloss, and A. D. Mcguire, "Comparing global models of terrestrial net primary productivity (NPP):global pattern and differentiation by major biomes," *Glob Chang. Biol*, vol. 5, no. S1, pp. 16–24, 2010.

[17] B. Liu, X. Chen, Q. Meng, H. Yang, and J. Van Wart, "Agricultural and Forest Meteorology Estimating maize yield potential and yield gap with agro-climatic zones in China — Distinguish irrigated and rainfed conditions," *Agric. For. Meteorol.*, vol. 239, pp. 108–117, 2017.

[18] Q. Zu, C. Mi, D. L. Liu, L. He, Z. Kuang, and Q. Fang, "Spatio-temporal distribution of sugarcane potential yields and yield gaps in," vol. 92, no. October 2017, pp. 72–83, 2018.

[19] Z. Xinke, L. Luo, and X. Xinliang, "Temporal and spatial variation characteristics of climate production potential of maize in China in recent 30 years," pp. 94–101, 2012.

[20] X. Zhang, "PE(possible evapotranspiration) index of vegetation and vegetation-climate classification (I) -- introduction to several main methods and PEP procedures," *J. plant Ecol.*, vol. 13, no. 1, pp. 197–207, 1989.

[21] "A preliminary study on the net primary productivity model of natural vegetation," *J. plant Ecol.*, vol. 19, no. 3, pp. 193–200, 1995.

[22] C. S. Potter *et al.*, "Terrestrial ecosystem production: A process model based on global satellite and surface data," *Global Biogeochem. Cycles*, vol. 7, no. 4, pp. 811–841, 1993.

[23] H. Lieth, *Modeling the Primary Productivity of the World*. 1975.

[24] Z. H. Chen *et al.*, "Estimation of Heihe Basin Net Primary Productivity Using the CASA Model," *J. Nat. Resour.*, 2008.

[25] K. Yin, Y. Tian, C. Yuan, F. Zhang, Q. Yuan, and L. Hua, "NPP spatial and temporal pattern of vegetation in Beijing and its factor explanation based on CASA model," *Remote Sens. L. Resour.*, vol. 27, no. 1, pp. 133–139, 2015.

[26] K. Chen, Y. Han, S. Cao, J. Ma, G. Cao, and H. Lu, "The Study of Vegetation Carbon Storage in Qinghai Lake Valley Based on Remote Sensing and CASA Model," *Procedia Environ. Sci.*, vol. 10, no. Part B, pp. 1568–1574, 2011.

[27] P. Shi-Long and Q. H. Guo, "APPLICATION OF CASA MODEL TO THE ESTIMATION OF CHINESE TERRESTRIAL NET PRIMARY PRODUCTIVITY," *Acta Phytoecol. Sin.*, vol. 25, no. 5, 2001.

[28] W. Zhu, Y. Pan, and J. Zhang, "Estimation of net primary productivity of chinese terrestrial vegetation based on remote sensing (In Chinese)," *Chinese J. Plant Ecol.*, vol. 31, no. 3, pp. 413–424, 2007.

[29] Q. Sun, C. Miao, and Q. Duan, "Extreme climate events and agricultural climate indices in China: CMIP5 model evaluation and projections," *Int. J. Climatol.*, vol. 36, no. 1, pp. 43–61, 2016.

[30] S. Chen *et al.*, "Evaluation of the successive V6 and V7 TRMM multisatellite precipitation analysis over the Continental United States," *Water Resour. Res.*, vol. 49, no. 12, pp. 8174–8186, 2013.

[31] H. Guo *et al.*, "Early assessment of Integrated Multi-satellite Retrievals for Global Precipitation Measurement over China," *Atmos. Res.*, vol. 176–177, pp. 121–133, 2016.

[32] P. Schubert *et al.*, "Remote Sensing of Environment Modeling GPP in the Nordic forest landscape with MODIS time series data — Comparison with the MODIS GPP product," *Remote Sens. Environ.*, vol. 126, pp. 136–147, 2012.

[33] D. P. Turner *et al.*, "Evaluation of MODIS NPP and GPP products across multiple biomes," vol. 102, pp. 282–292, 2006.

[34] C. Shim, J. Hong, J. Hong, Y. Kim, and M. Kang, "ScienceDirect Evaluation of MODIS GPP over a complex ecosystem in East Asia : A case study at Gwangneung flux tower in Korea," *Adv. Sp. Res.*, vol. 54, no. 11, pp. 2296–2308, 2014.

[35] T. Luo, "Biological productivity patterns and mathematical models of major forest types in China," 1996.

Formatted: Border: Bottom: (No border)

[36] J. He, P. Zhang, W. Jing, and Y. Yan, "Spatial Responses of Net Ecosystem Productivity of the Yellow River Basin under Diurnal Asymmetric Warming," *Sustainability*, vol. 10, no. 10, p. 3646, 2018.

[37] J. Li *et al.*, "Response of net primary production to land use and land cover change in mainland China since the late 1980s," *Sci. Total Environ.*, vol. 639, pp. 237–247, 2018.

[38] C. ming SUN, X. chun ZHONG, C. CHEN, T. GU, and W. CHEN, "Evaluating the grassland net primary productivity of southern China from 2000 to 2011 using a new climate productivity model," *J. Integr. Agric.*, vol. 15, no. 7, pp. 1638–1644, 2016.

[39] WeiZHOU, ZhengGuoSUN, JianLongLI, ChengChengGANG, and ChaoBinZHANG, "DesertificationdynamicandtherelativerolesofclimatechangeandhumanactivitiesindesertificationintheHeiheRiverBasinbasedonNPP," *J. Arid Land*, vol. 5, no. 4, pp. 465–479, 2013.

[40] F. Gu *et al.*, "Climate-driven uncertainties in modeling terrestrial ecosystem net primary productivity in China," *Agric. For. Meteorol.*, vol. 246, no. June, pp. 123–132, 2017.

[41] F. Fenga *et al.*, "Net primary productivity of China's terrestrial ecosystems from a process model driven by remote sensing," *J. Environ. Manage.*, vol. 85, pp. 563–573, 2007.

[42] F. Pei, X. Li, X. Liu, and C. Lao, "Assessing the impacts of droughts on net primary productivity in China," *J. Environ. Manage.*, vol. 114, pp. 362–371, 2013.

[43] W. Jiao, Y. Chen, W. Li, C. Zhu, and Z. Li, "Estimation of net primary productivity and its driving factors in the Ili River Valley, China," *J. Arid Land*, vol. 10, no. 5, pp. 781–793, 2018.

[44] W. Zhu, Y. Pan, and J. Zhang, "Remote sensing estimation of net primary productivity of terrestrial vegetation in China," *J. plant Ecol.*, vol. 31, no. 3, pp. 413–424, 2007.

[45] I. P. Holman, "A soil-based approach to rainfall-runoff modelling in ungauged catchments for England and Wales Supervisor," *Cranf. Univ.*, 2004.

[46] J. Zhao, X. Yan, J. Guo, and G. Jia, "Evaluating Spatial-Temporal Dynamics of Net Primary Productivity of Different Forest Types in Northeastern China Based on Improved FORCCHN," *PLoS One*, vol. 7, no. 11, pp. 1–14, 2012.

[47] D. P. Turner *et al.*, "Site-level evaluation of satellite-based global terrestrial gross primary production and net primary production monitoring," *Glob. Chang. Biol.*, vol. 11, no. 4, pp. 666–684, 2005.

Preprint PDF

Click here to access/download
Preprint PDF
Evaluation of NPP using three models compared over China .pdf
